# EEPD1 Inhibition Unleashes Antitumor Immunity in Colorectal Cancer by Activating the cGAS‐STING Pathway

**DOI:** 10.1002/advs.202522826

**Published:** 2026-03-30

**Authors:** Liyun Huo, Chong Wu, Xiaobo Li, Caina Ma, Jiamin Huang, Zishuo Chen, Weiling He, Tianyu Tao, Weijing Zhang

**Affiliations:** ^1^ Department of Gastrointestinal Surgery The First Affiliated Hospital Sun Yat‐sen University Guangzhou Guangdong China; ^2^ The Core Facilities for Medical Science at Zhongshan School of Medicine Sun Yat‐sen University Guangzhou Guangdong China; ^3^ Department of Immunology and Microbiology Zhongshan School of Medicine Sun Yat‐Sen University Guangzhou Guangdong China; ^4^ Department of Microbiology Zhongshan School of Medicine Sun Yat‐Sen University Guangzhou China; ^5^ Dermatology Hospital Southern Medical University Guangzhou Guangdong China; ^6^ Department of Gastrointestinal Surgery Xiang'an Hospital of Xiamen University School of Medicine Xiamen University Xiamen Fujian China; ^7^ Cancer Research Institute School of Basic Medical Sciences Southern Medical University Guangzhou China; ^8^ Department of Radiology State Key Laboratory of Oncology in South China Guangdong Provincial Clinical Research Center for Cancer Collaborative Innovation Center for Cancer Medicine Sun Yat‐sen University Cancer Center Guangzhou Guangdong China

**Keywords:** antitumor immunity, cGAS‐STING pathway, colorectal cancer, EEPD1, immunotherapy

## Abstract

The efficacy of immunotherapy in colorectal cancer (CRC) is frequently limited by an immunosuppressive tumor microenvironment. Here, we identify exonuclease/endonuclease/phosphatase domain‐containing protein 1 (EEPD1), a gatekeeper of homologous recombination (HR) repair, as a key driver of this immune exclusion. Downregulation of EEPD1 profoundly compromises HR in CRC cells, which curtails their proliferative and metastatic capacities in vitro and in vivo. Mechanistically, EEPD1 deficiency fuels genomic instability, leading to cytosolic DNA accumulation and activation of the cGAS‐STING‐type I interferon axis. This cascade promotes dendritic cell maturation, skews macrophages toward a proinflammatory M1 phenotype, and enhances tumor‐associated antigen presentation, culminating in robust CD8^+^ T cell activation and cytotoxicity. Our findings elucidate a direct mechanism by which targeting EEPD1 sensitizes CRC to anti‐PD1 immunotherapy. Our work establishes EEPD1 as a promising therapeutic target to overcome immune checkpoint blockade resistance in colorectal cancer.

## Graphical abstract

In colorectal cancer, high expression of the DNA repair protein EEPD1 correlates with immune exclusion and poor prognosis. This study demonstrates that EEPD1 depletion induces genomic instability, leading to cytosolic DNA accumulation and subsequent activation of the cGAS‐STING‐type I interferon pathway. This cascade remodels the tumor microenvironment by promoting dendritic cell maturation, M1 macrophage polarization, and CD8^+^ T cell‐mediated cytotoxicity, ultimately sensitizing tumors to anti‐PD1 immunotherapy and establishing EEPD1 as a promising therapeutic target.

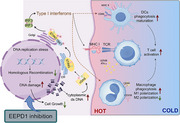

## 1 Introduction

Colorectal cancer (CRC) is a leading cause of cancer‐related mortality worldwide, with a bleak prognosis for patients with advanced disease [1, 2, 3]. While targeted therapies and immunotherapy have modestly improved outcomes, their efficacy is often transient due to substantial tumor heterogeneity and a profoundly immunosuppressive tumor microenvironment (TME) [4, 5, 6]. A key feature of this TME is diminished immunogenicity, characterized by inadequate antigen presentation and impaired T cell function, which presents a formidable barrier to effective immune checkpoint blockade (ICB) [7, 8, 9].

Genomic instability is a fundamental hallmark of cancer that can, paradoxically, be exploited for therapeutic benefit [10, 11, 12]. The DNA damage response (DDR) network is essential for maintaining genomic integrity, and its inhibition represents a compelling anticancer strategy [13, 14]. Targeting DDR pathways can synergize with conventional therapies and, critically, can remodel the TME to favor antitumor immunity [15, 16, 17, 18, 19, 20]. This is often achieved by inducing the accumulation of cytosolic double‐stranded DNA (dsDNA), which is sensed by the cyclic GMP‐AMP synthase (cGAS) [21, 22]. This engagement activates the stimulator of interferon genes (STING) pathway, leading to the production of type I interferons (IFNs) and the upregulation of interferon‐stimulated genes (ISGs) [23, 24]. This cascade can disrupt immune tolerance and enhance tumor cell recognition by the immune system, suggesting that targeting DDR is a promising approach to sensitize CRC to immunotherapy [25, 26, 27].

EEPD1 is a 5’ nuclease that functions as a critical gatekeeper for HR repair by facilitating DNA end resection at stalled replication forks and double‐strand breaks [28, 29]. Its role in cancer is context‐dependent; while implicated as an oncogene in esophageal and colorectal cancers [30, 31, 32], it can be protective in other malignancies [33, 34, 35]. Although bioinformatic studies have suggested EEPD1 as a prognostic biomarker in CRC [31, 32], its precise mechanistic contributions to CRC progression and, importantly, its influence on the tumor immune landscape remain unexplored. Whether EEPD1‐mediated DNA repair can be targeted to reshape the TME and augment immunotherapy efficacy has not been investigated.

In this study, we demonstrate that elevated EEPD1 expression in advanced CRC correlates with an immune‐excluded phenotype. We show that EEPD1 depletion not only suppresses tumor growth by impairing HR but also triggers a potent antitumor immune response. This response is driven by genomic instability‐induced activation of the cGAS‐STING‐type I IFN pathway, which promotes antigen presentation, enhances dendritic cell and macrophage function, and invigorates CD8^+^ T cells. Consequently, targeting EEPD1 synergizes with anti‐PD1 therapy, positioning it as a novel target for combination immunotherapy in CRC.

## 2 Results

### 2.1 EEPD1 is Upregulated in Advanced CRC and Correlates With an Immune‐ Excluded Microenvironment

**FIGURE 1:**
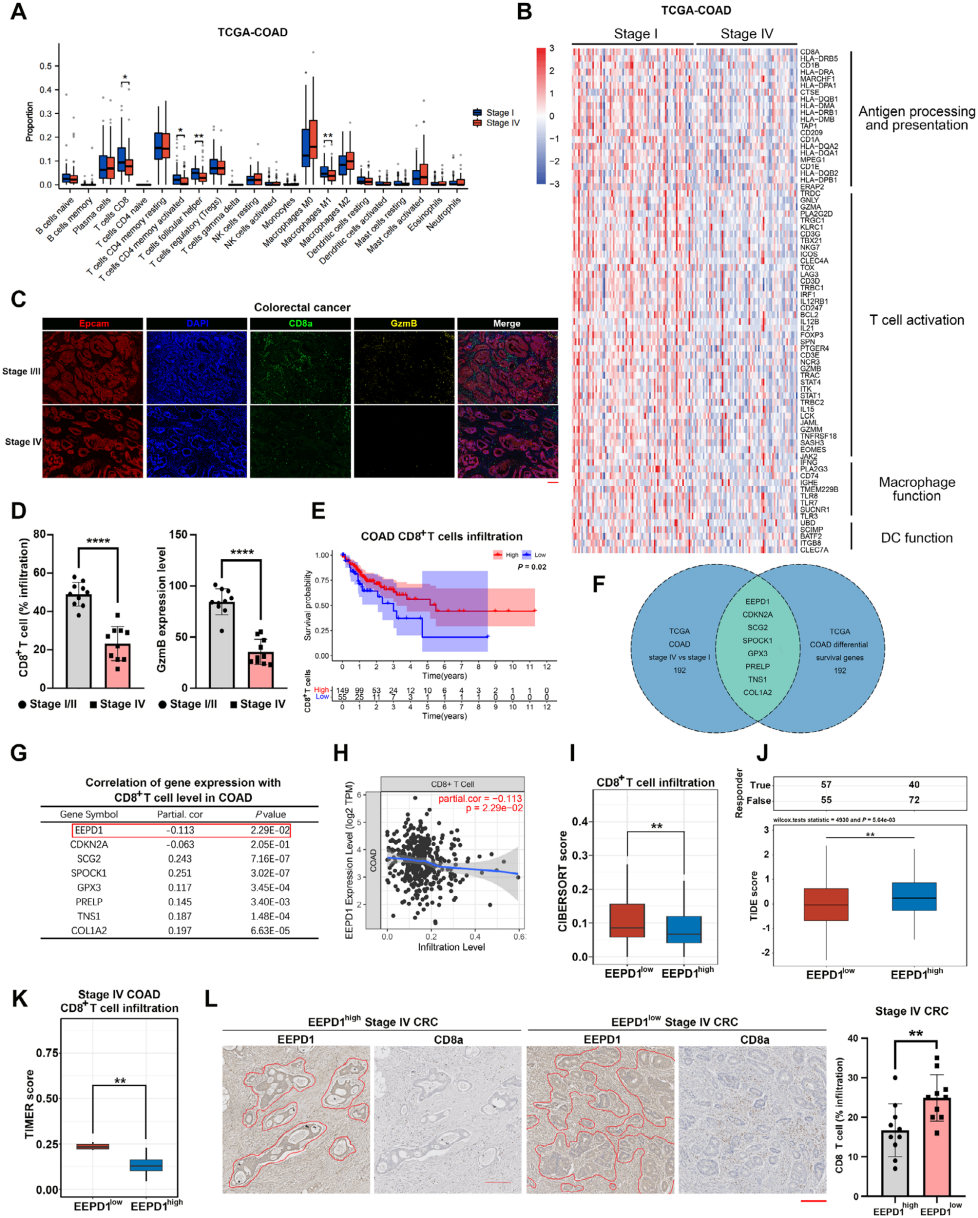
EEPD1 is upregulated in advanced CRC and correlates with an immune‐excluded microenvironment. (A) CIBERSORT analysis of immune cell infiltration in stage I vs. stage IV CRC patient tumors from the TCGA‐COAD dataset. (B) Heatmap depicting differentially expressed immune‐related genes between stage I and stage IV tumors. Gene Ontology (GO) enrichment analysis of immune‐related pathways is shown on the right. (C) Representative immunofluorescence images of CD8 (green) and Granzyme B (GZMB, red) in early‐stage (I/II) and advanced‐stage (IV) CRC patient tissues. Nuclei are stained with DAPI (blue). Scale bar, 200 µm. (D) Quantification of CD8^+^ and GZMB^+^ cells as a percentage of total cells from (C), (Sample size *n* = 10). (E) Kaplan‐Meier analysis of overall survival in the TCGA‐COAD cohort stratified by high vs. low CD8^+^ T cell infiltration. (F) Venn diagram illustrating the intersection of genes upregulated in late‐stage CRC (TCGA) and genes associated with poor prognosis in COAD (GEPIA). (G) Correlation analysis between the eight intersecting genes and CD8^+^ T cell infiltration in COAD from the TIMER database. (H) Scatter plot from TIMER showing the negative correlation between EEPD1 expression and CD8^+^ T cell infiltration. (I) CIBERSORT analysis showing the correlation between EEPD1 expression and CD8^+^ T cell abundance in all COAD patients. (J) TIDE prediction scores for immunotherapy response in COAD patients stratified by EEPD1 expression. (K) TIMER analysis revealing the correlation between EEPD1 expression and CD8^+^ T cell abundance in stage IV COAD patients exclusively. (L) Representative immunohistochemistry (IHC) images of EEPD1 and CD8 staining on serial sections from advanced CRC patient tumors. (Sample size *n* = 10), Scale bar, 200 µm. Quantification of staining intensity is shown on the right. Data are presented as mean ± SD. Statistical analysis was performed using an unpaired two‐tailed Student's *t*‐test (A, D, I, J, K, L), Spearman's rank correlation (G, H), and the log‐rank test (E). ^*^ *p* < 0.05, ^**^ *p* < 0.01, ^****^ *p* < 0.0001.

To define the immunological landscape of advanced colorectal cancer, we first analyzed immune cell infiltration in the TCGA‐COAD dataset using CIBERSORT. This revealed that stage IV tumors harbored significantly fewer cytotoxic CD8^+^ T cells, activated CD4^+^ memory T cells, and M1 macrophages compared to stage I tumors (Figure 1A). Gene expression analysis further demonstrated a widespread downregulation of pathways related to antigen processing and presentation, T cell activation, and dendritic cell (DC) function in stage IV patients (Figure 1B). To validate these findings, we performed immunofluorescence staining on primary tumor sections from our institutional cohort, which confirmed a marked reduction in CD8^+^ T cell infiltration and granzyme B (GZMB) expression in advanced lesions (Figure 1C,D). Consistent with this, low CD8^+^ T cell infiltration in the TCGA‐COAD cohort was associated with shorter overall survival (Figure 1E).

To identify regulators of this immune‐excluded phenotype, we intersected the top 200 genes upregulated in stage IV CRC with the 200 genes most strongly correlated with poor prognosis, yielding eight candidates (Figure 1F). Subsequent correlation analysis using TIMER 2.0 indicated that among these candidates, only EEPD1 expression showed a significant negative correlation with CD8^+^ T cell infiltration in CRC (Figure 1G,H). Further analysis confirmed that high EEPD1 expression was associated with diminished CD8^+^ T cell infiltration and a lower predicted response to immune checkpoint blockade (ICB) (Figure 1I,J). We then confirmed that EEPD1 mRNA and protein levels were significantly elevated in CRC tissues compared to adjacent normal mucosa and were associated with shorter overall and disease‐free survival (Figure S1A–E).

Notably, EEPD1 expression was progressively higher in advanced‐stage tumors (Figure S1F,G). Moreover, EEPD1 was significantly upregulated in matched lymph node and liver metastases compared to primary tumors (Figure S1H). Furthermore, TIMER analysis revealed that CD8^+^ T cell infiltration was significantly reduced in stage IV CRC patients with high EEPD1 expression (Figure 1K). Finally, the immunohistochemistry on serial sections verified the stark inverse relationship: tumors with high EEPD1 expression displayed significantly reduced CD8^+^ T cell infiltration in stage IV CRC patients (Figure 1L; Figure S1I). Collectively, these data identify EEPD1 as a clinically relevant protein whose upregulation is a feature of an immunosuppressive microenvironment in advanced CRC.

### 2.2 EEPD1 Depletion Suppresses CRC Proliferation and Metastasis

**FIGURE 2:**
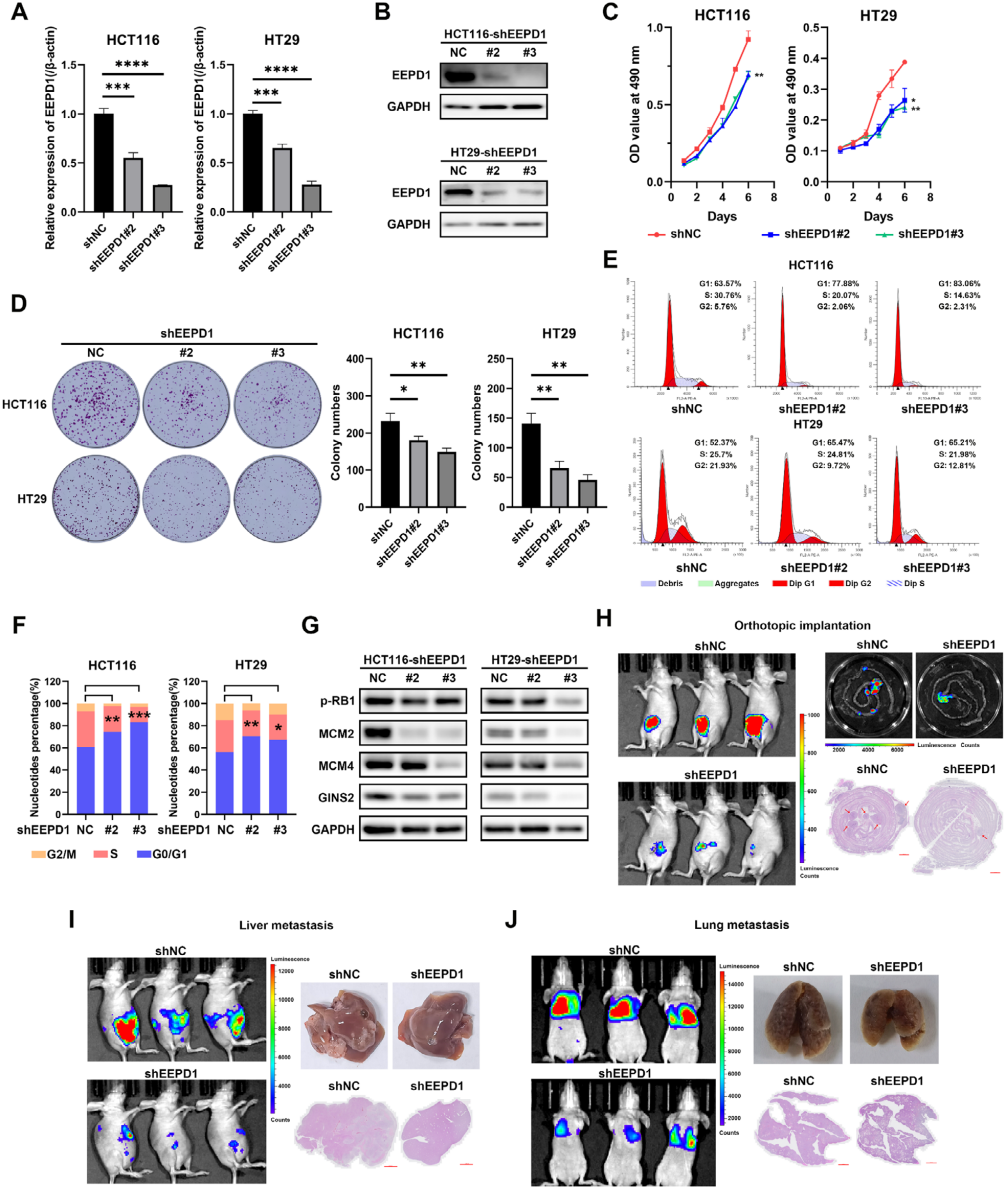
EEPD1 depletion suppresses CRC proliferation and metastasis. (A,B) RT‐qPCR (A) and western blot (B) analysis of EEPD1 expression in HCT116 and HT29 cells stably expressing control (shNC) or EEPD1‐targeting shRNA (shEEPD1). (C) Proliferation of shNC and shEEPD1 HCT116 and HT29 cells assessed by MTT assay over 144 h. (D) Colony formation capacity of shNC and shEEPD1 HCT116 and HT29 cells. Representative images and quantification are shown. (E,F) Cell cycle distribution of shNC and shEEPD1 HCT116 (E) and HT29 (F) cells analyzed by flow cytometry after propidium iodide staining. (G) Western blot analysis of key cell cycle regulatory proteins in shNC and shEEPD1 cells. (H–J) In vivo assessment of tumor growth and metastasis. (H) Orthotopic model showing bioluminescence imaging of tumors in situ (left), ex vivo (top right), and H&E staining of tumor sections (bottom right) (*n* = 3 mice per group). (I) Liver metastasis model showing bioluminescence imaging (left), ex vivo liver images (top right), and H&E staining of metastatic nodules (bottom right) (*n* = 3 mice per group). (J) Lung metastasis model showing bioluminescence imaging (left), ex vivo lung images (top right), and H&E staining of metastatic nodules (bottom right) (*n* = 3 mice per group). Scale bars, 2 mm. Data are presented as mean ± SD from three independent experiments. Statistical analysis was performed using one‐way ANOVA (A, D, F) and two‐way ANOVA (C). ^*^ *p* < 0.05, ^**^ *p* < 0.01, ^***^ *p* < 0.001, ^****^ *p* < 0.0001.

To investigate the functional role of EEPD1 in CRC, we established stable knockdown models in human (HCT116, HT29) and murine (MC38, CT26) CRC cell lines, confirming efficient downregulation at both the mRNA and protein levels (Figure 2A,B; Figure S2A,B). Functional assays demonstrated that EEPD1 deficiency severely impaired cellular proliferation, as evidenced by suppressed growth in MTT assays and reduced colony formation ability (Figure 2C,D; Figure S2C,D). Cell cycle analysis revealed that EEPD1 depletion induced a G1/S phase arrest, characterized by a significant reduction in the S‐phase population (Figure 2E,F; Figure S2E,F). This cell cycle blockade was accompanied by the downregulation of key regulatory proteins, including p‐RB1, MCM2, MCM4, and GINS2 (Figure 2G; Figure S2G). We further established EEPD1 knockdown in NCM460 human normal colonic epithelial cells (Figure S2H). Although EEPD1 inhibition significantly suppressed the growth of NCM460 cells in MTT assays, the inhibitory effect was less pronounced than that observed in HCT116 colorectal cancer cells (Figure S2I,J). This differential sensitivity suggests a potential therapeutic window for EEPD1‐targeted therapy in colorectal cancer. We next assessed the impact of EEPD1 loss in vivo. In an orthotopic CRC model, tumors derived from EEPD1‐knockdown cells exhibited significantly attenuated growth compared to controls (Figure 2H). Given the association of high EEPD1 with advanced disease, we evaluated its role in metastasis. In both splenic injection‐induced liver metastasis and tail vein‐induced lung metastasis models, EEPD1 depletion dramatically reduced the metastatic burden, as measured by in vivo imaging and confirmed by histological analysis (Figure 2I,J). These results establish that EEPD1 is a critical driver of CRC proliferation and metastatic dissemination.

### 2.3 EEPD1 Loss Remodels the Tumor Immune Microenvironment to Favor Antitumor Immunity

**FIGURE 3:**
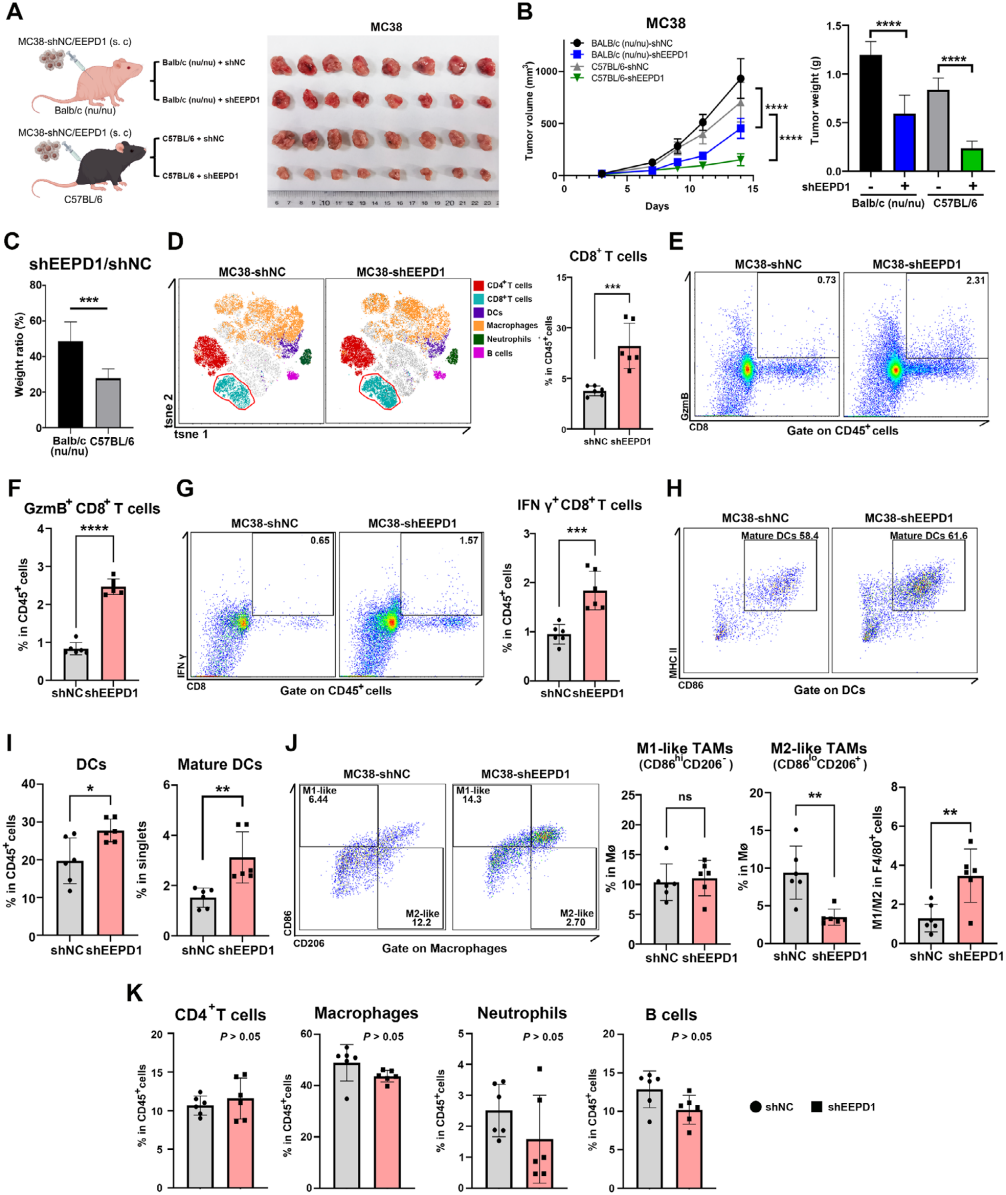
EEPD1 loss remodels the tumor immune microenvironment to favor antitumor immunity. (A) Representative images of subcutaneous MC38‐shNC and MC38‐shEEPD1 tumors grown in immunodeficient (BALB/c nu/nu) and immunocompetent (C57BL/6) mice (*n* = 8 mice per group). (B) Tumor growth curves and final tumor volumes for the experiment described in (A). (C) Tumor weight ratio (shEEPD1/shNC) in immunodeficient vs. immunocompetent mice, indicating immune‐mediated tumor control. (D) t‐SNE visualization of the immune landscape in MC38 tumors from C57BL/6 mice, analyzed by spectral flow cytometry. Quantification of the CD8^+^ T cell population (CD45^+^CD3^+^CD8^+^) is shown. (*n* = 6 mice per group) (E–G) Flow cytometric analysis of effector molecule expression in tumor‐infiltrating CD8^+^ T cells. Representative plots (E) and quantification of GZMB^+^ (F) and IFN‐γ^+^ (G) CD8^+^ T cells. (H,I) Analysis of dendritic cell (DC) maturation. Representative plots (H) and quantification of mature DCs (CD11c^+^MHC‐II^+^CD86^+^) (I). (J) Analysis of macrophage polarization, showing the ratio of M1 (F4/80^+^CD86^+^CD206^−^) to M2 (F4/80^+^CD86^−^CD206^+^) macrophages. (K) Quantification of tumor‐infiltrating CD4^+^ T cells, total macrophages, neutrophils, and B cells. Data are presented as mean ± SD (*n* = 8 mice per group for A‐C; or *n* = 6 mice per group for D–K). Statistical analysis was performed using two‐way ANOVA (B, tumor volume curve), one‐way ANOVA (B, tumor weight), and an unpaired two‐tailed Student's *t*‐test (C–K). ^*^ *p* < 0.05, ^**^ *p* < 0.01, ^***^ *p* < 0.001, ^****^ *p* < 0.0001.

Building on our findings that EEPD1 knockdown suppresses tumor growth and is associated with immune exclusion, we hypothesized that the antitumor effect is partly immune‐mediated. To test this, we established subcutaneous tumors in both immunodeficient BALB/c (nu/nu) and immunocompetent C57BL/6 or BALB/c mice. While EEPD1 knockdown suppressed tumor growth in all models without changes in mice body weight, the effect was markedly more potent in immunocompetent hosts (Figure 3A–C; Figure S3A–D), strongly suggesting the involvement of an intact immune system.

To characterize the immunological changes, we performed spectral flow cytometry on dissociated tumors from immunocompetent mice. In both microsatellite instability (MC38) and microsatellite stable (CT26) tumor models, EEPD1 depletion led to a significant increase in the infiltration of CD8^+^ T cells (Figure 3D; Figure S3E). These tumor‐infiltrating CD8^+^ T cells displayed enhanced effector function, with elevated expression of GZMB and interferon‐gamma (IFN‐γ) (Figure 3E–G; Figure S3F,G). Furthermore, EEPD1‐knockdown tumors exhibited increased infiltration of DCs and a higher proportion of mature CD86^+^MHC‐II^+^ DCs in singlets (Figure 3H,I; Figure S3H), suggesting DC involvement in the immune activation process. While the overall macrophage infiltration was unchanged, their polarization was shifted toward a proinflammatory state, reflected by an increased M1/M2 ratio (Figure 3J; Figure S3I,J). In contrast, no significant changes were observed in CD4^+^ T cells, neutrophils, or B cells (Figure 3K; Figure S3K). Together, these data demonstrate that EEPD1 loss reverses immune tolerance by fostering a T cell‐inflamed microenvironment rich in activated effector cells.

### 2.4 EEPD1 Deficiency Enhances MHC‐I Antigen Presentation and Sensitizes CRC Cells to T Cell Killing

**FIGURE 4:**
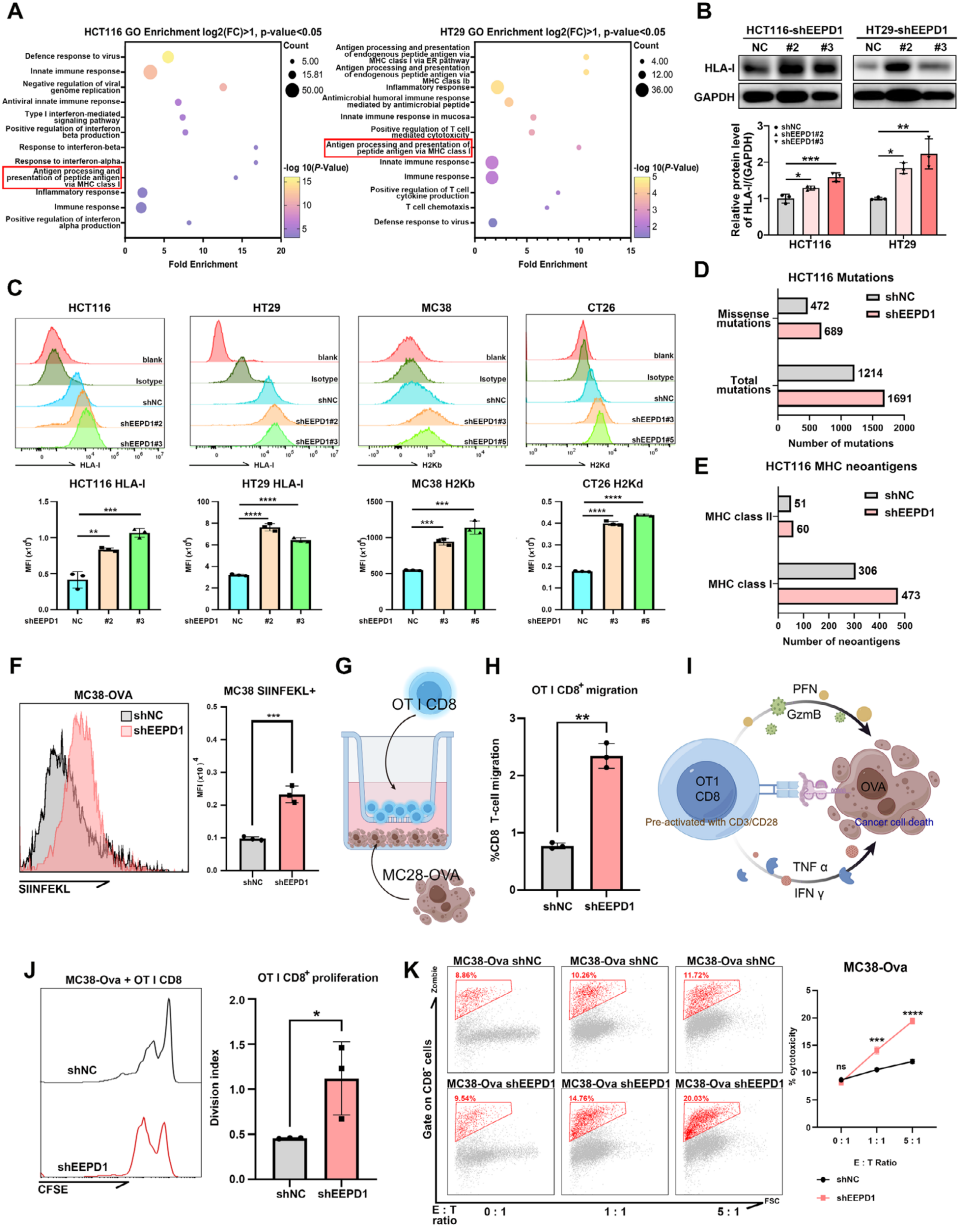
EEPD1 deficiency enhances MHC‐I antigen presentation and sensitizes CRC cells to T cell killing. (A) GO enrichment analysis of upregulated immune‐related pathways from RNA‐seq data of EEPD1‐knockdown HCT116 and HT29 cells. (B) Western blot analysis of HLA‐I protein levels in HCT116 and HT29 cells. (C) Flow cytometric analysis of surface MHC‐I expression on human (HCT116, HT29) and murine (MC38, CT26) CRC cell lines. (D) Whole‐exome sequencing (WES) analysis showing the number of newly emerged missense mutations and total somatic mutations in HCT116‐shEEPD1 cells compared to shNC controls. (E) Number of predicted high‐affinity MHC‐I and MHC‐II binding neoantigens derived from WES data. (F) Flow cytometric analysis of surface SIINFEKL‐H2‐K^b^ complex on MC38‐OVA cells. (G, H) OT‐I CD8^+^ T cell migration assay in a two‐chamber system with MC38‐OVA cells. Schematic diagram (G) and quantification of the T cell migration rate (H) were shown. (I,J) OT‐I CD8^+^ T cell proliferation assay. CFSE‐labeled T cells were co‐cultured with MC38‐OVA cells (E:T ratio 5:1) for 72 h. Representative schematic diagram (I) and quantification of the division index (J) are shown. (K) T cell‐mediated cytotoxicity assay. MC38‐OVA cells were co‐cultured with OT‐I T cells at indicated E:T ratios for 24 h. Tumor cell death (CD45^−^Zombie Aqua^+^) was quantified by flow cytometry. Data are presented as mean ± SD from three independent experiments (*n* = 3). Statistical analysis was performed using one‐way ANOVA (B, C) and an unpaired two‐tailed Student's *t*‐test (F,H,J,K). ^*^ *p* < 0.05, ^**^ *p* < 0.01, ^***^ *p* < 0.001, ^****^ *p* < 0.0001.

Given the immune activation observed upon EEPD1 inhibition in colorectal cancer, we initially hypothesized that changes in PD‐L1 expression might underlie this effect. To test this, we examined PD‐L1 levels in EEPD1‐knockdown cells. Although total PD‐L1 protein expression remained largely unchanged, flow cytometry revealed either increased or unaltered surface expression—findings inconsistent with the enhanced anti‐tumor immunity we previously demonstrated (Figure S4A,B). Furthermore, analysis of PD‐L1 expression in primary colorectal cancer lesions showed no significant correlation with EEPD1 levels (Figure S4C,D). To uncover the molecular mechanisms driving this immune activation, we performed RNA sequencing on EEPD1‐knockdown cells. Gene Ontology analysis revealed that “antigen processing and presentation via MHC class I” was among the most significantly enriched immune‐related pathways in both HCT116 and HT29 cells (Figure 4A). We validated this finding at the protein level, observing a marked increase in HLA‐I expression by immunoblotting and stronger membrane localization by immunofluorescence (Figure 4B; Figure S4E,F). Flow cytometry confirmed a sustained upregulation of surface MHC‐I across multiple CRC lines upon EEPD1 knockdown (Figure 4C).

Given EEPD1's role in HR repair, we hypothesized that its loss would increase genomic instability and neoantigen generation. Whole‐exome sequencing of EEPD1‐knockdown HCT116 cells revealed a higher mutational load, including an increase in missense mutations, nonsynonymous single nucleotide variants (SNVs), and frameshift indels, resulting in a greater tumor mutational burden (Figure 4D; Figure S4G,H). This translated to a higher predicted number of both MHC‐I‐ and MHC‐II‐binding neoantigens (Figure 4E; Figure S4I). To test the functional consequence of these changes, we used MC38 cells expressing ovalbumin (MC38‐OVA). EEPD1 knockdown enhanced the surface presentation of the cognate SIINFEKL peptide (Figure 4F). In a two‐chamber system, EEPD1‐downregulated MC38‐OVA cells induced migration of activated CD8^+^ T cells (Figure 4G,H). In co‐culture assays, EEPD1‐deficient MC38‐OVA cells triggered significantly greater proliferation of OT‐I CD8^+^ T cells and were more susceptible to T cell‐mediated killing (Figure 4I–K). These results indicate that EEPD1 depletion enhances tumor immunogenicity by increasing both antigen abundance and the machinery for its presentation.

### 2.5 EEPD1 Inhibition Potentiates CD8^+^ T Cell Activation Through DC and Macrophage Crosstalk

**FIGURE 5:**
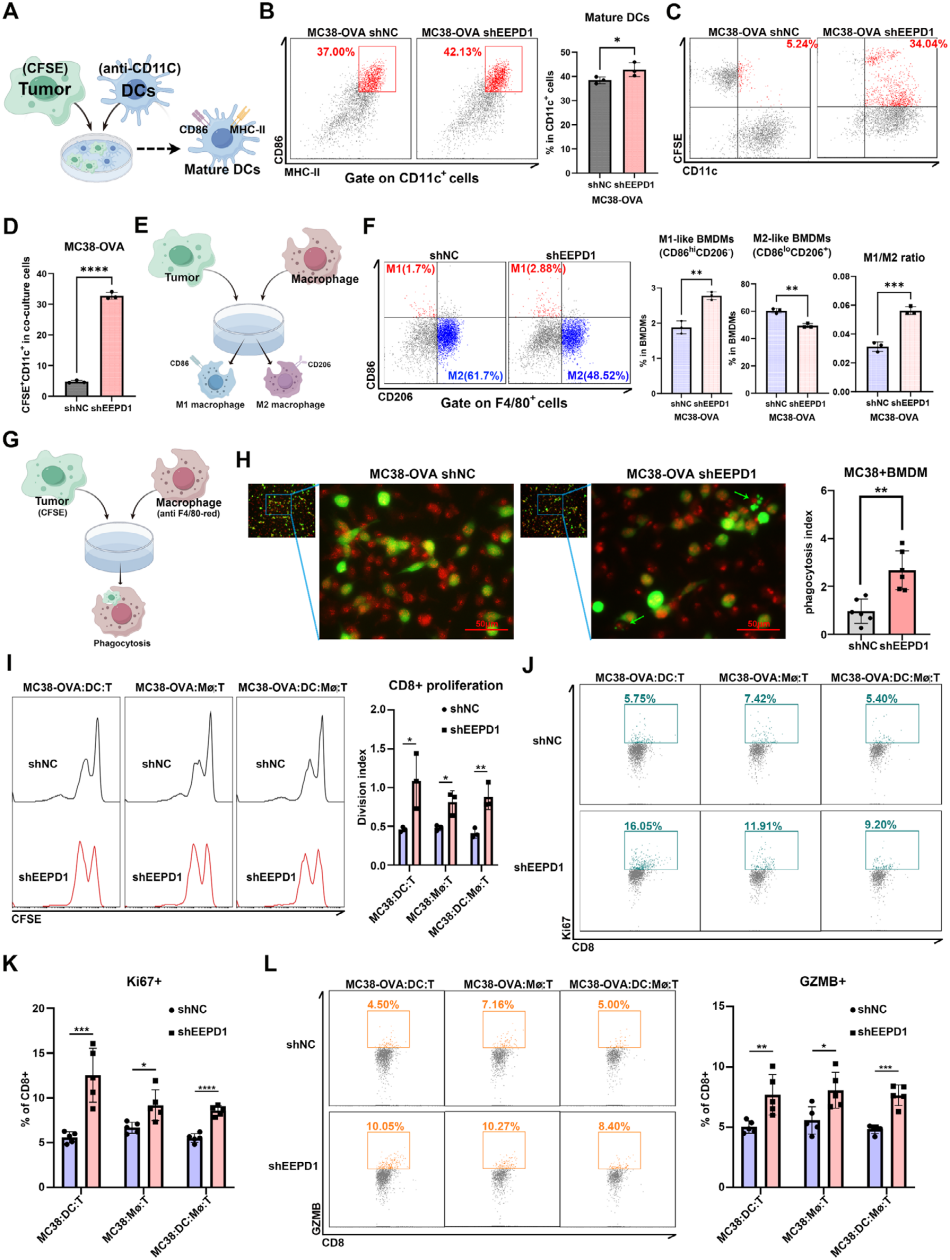
EEPD1 inhibition potentiates CD8^+^ T cell activation through DC and macrophage crosstalk. (A–D) Analysis of DC function. MC38‐OVA cells were co‐cultured with OT‐I bone marrow‐derived dendritic cells (BMDCs). (A,B) BMDC maturation (CD11c^+^MHC‐II^+^CD86^+^) was assessed by flow cytometry. (C,D) Phagocytosis of CFSE‐labeled tumor cells by BMDCs (CD11c^+^CFSE^+^) was quantified. (E,F) Analysis of macrophage function. MC38‐OVA cells were co‐cultured with OT‐I bone marrow‐derived macrophages (BMDMs). The M1/M2 polarization ratio was assessed by flow cytometry. Sample size *n* = 3. (G,H) Phagocytosis of CFSE‐labeled tumor cells by BMDMs. Representative immunofluorescence images (G) and flow cytometric quantification (H) are shown. Scale bar, 50 µm. Sample size *n* = 5. (I) T cell proliferation in a tripartite co‐culture system. CFSE‐labeled OT‐I CD8^+^ T cells were co‐cultured with MC38‐OVA cells and either BMDCs, BMDMs, or both. Proliferation was assessed by CFSE dilution. (J–L) T cell activation in the tripartite co‐culture. Expression of Ki‐67 (J,K) and GZMB (L) in OT‐I CD8^+^ T cells was measured by intracellular flow cytometry. Sample size *n* = 5. Data are presented as mean ± SD from three independent experiments (*n* = 3 for B–F, I; *n* = 6 for H; or *n* = 5 for J–L). Statistical analysis was performed using an unpaired two‐tailed Student's *t*‐test (B,D,F,H,I,K,L). ^*^ *p* < 0.05, ^**^ *p* < 0.01, ^***^ *p* < 0.001, ^****^ *p* < 0.0001.

We next investigated whether the observed changes in DCs and macrophages contribute to enhanced T cell activation. Co‐culturing bone marrow‐derived DCs (BMDCs) with EEPD1‐knockdown MC38‐OVA cells promoted DC maturation, as evidenced by increased CD86 and MHC‐II expression (Figure 5A,B; Figure S5A,B). These BMDCs also exhibited enhanced phagocytic uptake of EEPD1‐deficient tumor cells (Figure 5C,D). Similarly, co‐culture with bone marrow‐derived macrophages (BMDMs) skewed their polarization toward an M1 phenotype and increased the M1/M2 ratio (Figure 5E,F). BMDMs also displayed greater phagocytosis of EEPD1‐knockdown cells (Figure 5G,H; Figure S5C).

To determine the collective impact on T cell function, we established a tripartite co‐culture system. The presence of EEPD1‐knockdown tumor cells, together with BMDCs and/or BMDMs, led to a profound increase in OT‐I CD8^+^ T cell proliferation and activation, as measured by Ki‐67 and GZMB expression (Figure 5I–L). Notably, activated CD8^+^ T cells appeared to amplify this response, as their presence further enhanced DC maturation and M1 macrophage polarization (Figure S5D,E), suggesting a positive feedback loop that sustains antitumor immunity.

### 2.6 Type I Interferon Signaling is Essential for the Antitumor Immunity Induced by EEPD1 Loss

**FIGURE 6:**
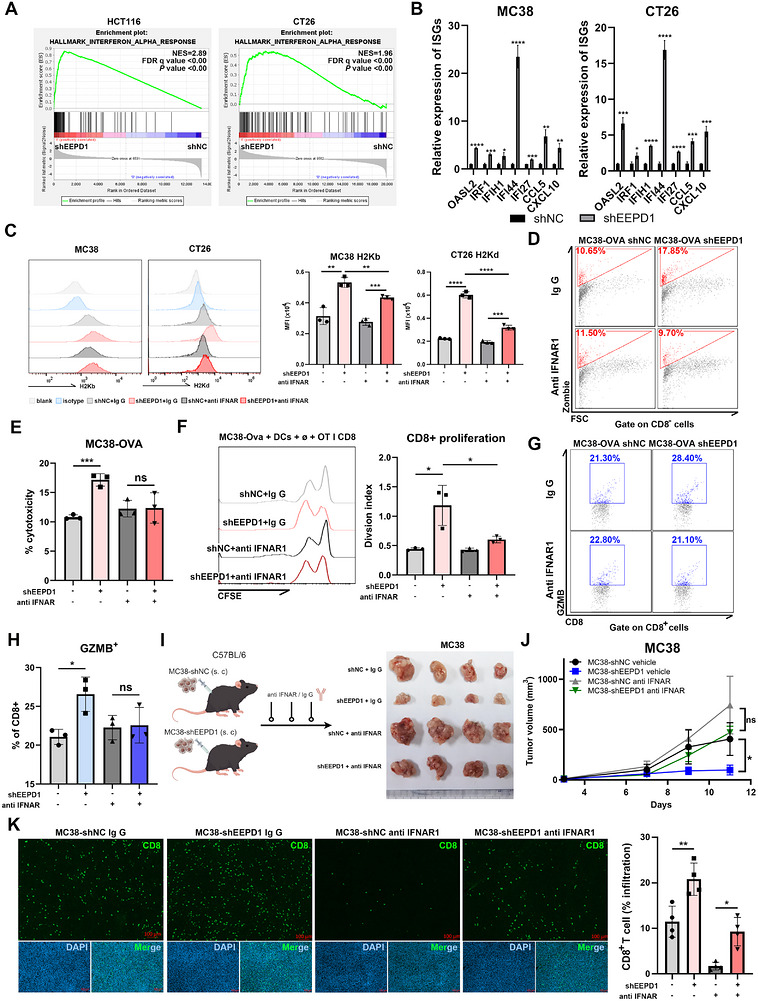
Type I interferon signaling is essential for the antitumor immunity induced by EEPD1 loss. (A) GSEA plots showing enrichment of the Hallmark_Interferon_Alpha_Response pathway in EEPD1‐knockdown HCT116 and CT26 cells. (B) RT‐qPCR analysis of ISG expression in EEPD1‐knockdown MC38 and CT26 cells. (C) Flow cytometric analysis of surface MHC‐I expression on EEPD1‐knockdown cells treated with an IFNAR1 blocking antibody (20 µg/mL) for 48 h. (D,E) T cell cytotoxicity assay. MC38‐OVA cells pre‐treated with anti‐IFNAR1 were co‐cultured with OT‐I T cells (E:T ratio 5:1). Tumor cell death was quantified by flow cytometry. (F) T cell proliferation assay. OT‐I T cells were co‐cultured with anti‐IFNAR1‐pretreated MC38‐OVA cells. Proliferation was assessed by CFSE dilution. (G,H) T cell activation assay. GZMB expression in OT‐I T cells co‐cultured with anti‐IFNAR1‐pretreated tumor cells was measured by flow cytometry. (I–K) In vivo IFNAR1 blockade. C57BL/6 mice bearing MC38‐shEEPD1 tumors were treated with anti‐IFNAR1 or isotype control antibody. (I) Representative tumor images. (J) Tumor growth curves. (K) Immunofluorescence staining and quantification of CD8^+^ T cell infiltration in tumor sections. Scale bar, 100 µm. Data are presented as mean ± SD from three independent experiments (*n* = 3 for in vitro experiments B–H; *n* = 4 mice per group for in vivo experiments I–K). Statistical analysis was performed using an unpaired two‐tailed Student's *t*‐test (B), one‐way ANOVA (C,E,F,H,K), and two‐way ANOVA (J). ^*^ *p* < 0.05, ^**^ *p* < 0.01, ^***^ *p* < 0.001, ^****^ *p* < 0.0001.

To identify the signaling pathway responsible for these immunological changes, we performed GSEA on our RNA‐seq data, which revealed a significant enrichment of the HALLMARK_INTERRERON_ALPHA_RESPONSE pathway in EEPD1‐knockdown cells (Figure 6A). This was confirmed by RT‐qPCR, which showed elevated expression of multiple interferon‐stimulated genes (ISGs) (Figure 6B; Figure S6A). Type I interferon pathway activation in tumor cells was reported to potentiate the antitumor functions of various immune cells within the TIME [36, 37, 38]. To test the functional necessity of this pathway, we used an anti‐IFNAR1 antibody to block type I IFN signaling. IFNAR1 blockade partially reversed the MHC‐I upregulation on EEPD1‐knockdown tumor cells and markedly reduced their susceptibility to T cell‐mediated killing (Figure 6C–E).

Furthermore, pre‐treatment of tumor cells with anti‐IFNAR1 attenuated their ability to promote DC maturation and M1 macrophage polarization in co‐culture (Figure S6B,C). In the tripartite co‐culture system, IFNAR1 blockade on tumor cells blunted both the proliferation and GZMB expression of CD8^+^ T cells (Figure 6F–H). Crucially, in vivo treatment with anti‐IFNAR1 completely abrogated the tumor growth inhibition conferred by EEPD1 knockdown and reduced CD8^+^ T cell infiltration in tumors (Figure 6I–K; Figure S6D,E). These results firmly establish that tumor cell‐intrinsic type I IFN signaling is the central driver of the immune‐mediated antitumor effects of EEPD1 inhibition.

### 2.7 EEPD1 Depletion Activates the cGAS‐STING Pathway via Induction of Genomic Instability

**FIGURE 7:**
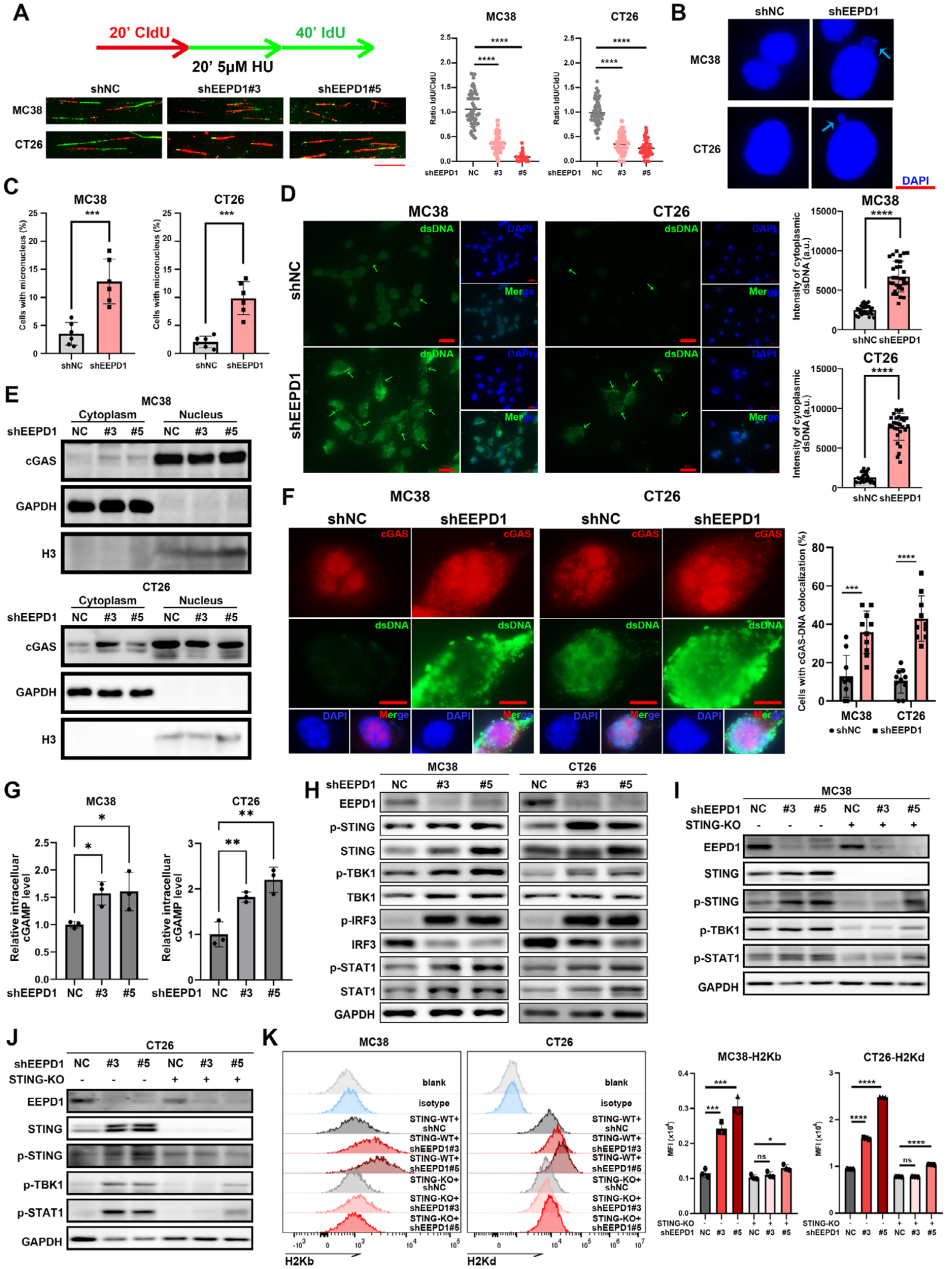
EEPD1 depletion activates the cGAS‐STING pathway via induction of genomic instability. (A) DNA fiber assay in MC38 and CT26 cells. The ratio of IdU to CldU tract length was measured to assess replication fork stability. Representative images and quantification are shown. Scale bar, 10 µm. (B,C) DAPI staining showing micronucleus formation in EEPD1‐knockdown cells. Scale bar, 10 µm. (D) Immunofluorescence staining and quantification of cytosolic dsDNA in EEPD1‐knockdown cells. Scale bars, 20 µm. (E) Western blot analysis of cGAS in cytoplasmic and nuclear fractions of EEPD1‐knockdown cells. Histone H3 and GAPDH served as nuclear and cytoplasmic markers, respectively. (F) Confocal microscopy showing co‐localization of cGAS (green) and dsDNA (red) in the cytoplasm of EEPD1‐knockdown cells. Scale bars, 5 µm. (G) ELISA quantification of cGAMP levels in cell lysates. (H) Western blot analysis of key proteins in the cGAS‐STING signaling pathway, including validation of EEPD1 knockdown efficiency. (I,J) Western blot analysis confirming that STING knockout (KO) abrogates downstream signaling activation in EEPD1‐knockdown cells. (K) Flow cytometric analysis showing that STING knockout reverses the MHC‐I upregulation induced by EEPD1 knockdown. Data are presented as mean ± SD from three independent experiments (*n* = 60 for A; *n* = 6 for C; *n* = 30 for D; *n* = 10 for F; *n* = 3 for G,K). Statistical analysis was performed using one‐way ANOVA (A, G, K) and an unpaired two‐tailed Student's *t*‐test (C,D,F). ^*^ *p* < 0.05, ^**^ *p* < 0.01, ^***^ *p* < 0.001, ^****^ *p* < 0.0001.

We next sought to determine how EEPD1 loss activates the type I IFN pathway. As a gatekeeper of HR repair, its deficiency is expected to cause genomic instability [28, 29]. Indeed, EEPD1‐knockdown cells displayed significantly reduced HR repair efficiency (Figure S7A) and compromised replication fork restart under stress, as shown by DNA fiber assays (Figure 7A; Figure S7B). This genomic instability manifested as a dramatic increase in micronuclei formation (Figure 7B,C; Figure S7C), which are known to rupture and release DNA into the cytoplasm [39]. Immunofluorescence staining confirmed a substantial accumulation of cytosolic dsDNA in EEPD1‐deficient cells (Figure 7D; Figure S7D,E).

This accumulation of cytosolic dsDNA triggered activation of the cGAS‐STING pathway [21, 40]. Immunoblotting and immunofluorescence revealed increased cytoplasmic localization of cGAS, where it co‐localized with dsDNA (Figure 7E,F). This engagement led to a marked increase in the production of the second messenger cyclic GMP‐AMP (cGAMP), as measured by ELISA (Figure 7G; Figure S7G). Consequently, we observed elevated phosphorylation of the downstream effectors STING, TBK1, IRF3, and STAT1, indicating robust pathway activation (Figure 7H; Figure S7H). To definitively link this cascade, we generated STING‐knockout (KO) cells. STING ablation completely reversed the EEPD1‐knockdown‐induced phosphorylation of downstream targets and abrogated the upregulation of surface MHC‐I (Figure 7I–K). These data demonstrate that EEPD1 deficiency activates type I IFN signaling by inducing genomic instability, which fuels the cGAS‐STING pathway.

### 2.8 Targeting EEPD1 Synergizes With Anti‐PD1 Immunotherapy to Control Tumor Growth

**FIGURE 8:**
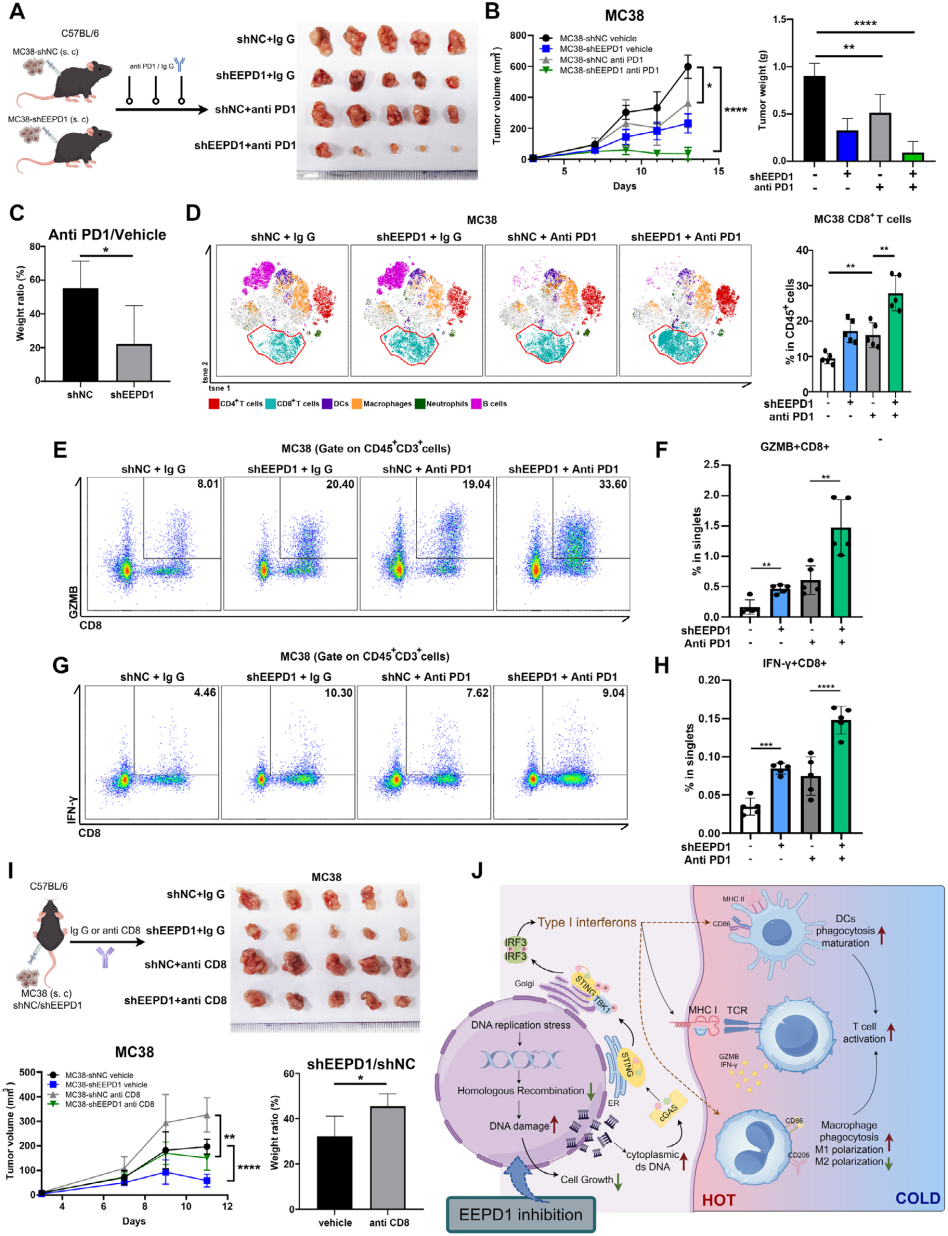
Targeting EEPD1 synergizes with anti‐PD1 immunotherapy to control tumor growth. (A–C) C57BL/6 mice bearing subcutaneous MC38 tumors were treated with anti‐PD1 or isotype control antibody. (A) Representative tumor images at the endpoint. (B) Tumor growth curves and final tumor volumes. (C) Tumor weight ratio (anti‐PD1/IgG). (D) Flow cytometric quantification of tumor‐infiltrating CD8^+^ T cells in the different treatment groups. (E–H) Analysis of CD8^+^ T cell effector function. Representative plots and quantification of GZMB^+^ (E,F) and IFN‐γ^+^ (G, H) CD8^+^ T cells. (I) CD8^+^ T cell depletion experiment. Mice bearing MC38 tumors were treated with an anti‐CD8α depleting antibody or isotype control. Tumor growth curves and final tumor weight ratio (shEEPD1/shNC) are shown. (J) Schematic model illustrating how EEPD1 inhibition remodels the tumor immune microenvironment to enhance immunotherapy efficacy. EEPD1 loss leads to genomic instability and cytosolic dsDNA accumulation, which activates the cGAS‐STING‐IFN‐I axis, thereby promoting DC maturation, M1 macrophage polarization, and CD8^+^ T cell‐mediated tumor killing. Data are presented as mean ± SD (*n* = 5 mice per group). Statistical analysis was performed using two‐way ANOVA (B,I), one‐way ANOVA (B,D,F,H), and an unpaired two‐tailed Student's *t*‐test (C,I). ^*^ *p* < 0.05, ^**^ *p* < 0.01, ^***^ *p* < 0.001, ^****^ *p* < 0.0001.

Given that EEPD1 depletion remodels the TME to be more T cell‐inflamed, we hypothesized it would sensitize tumors to anti‐PD1 therapy. We treated mice bearing established subcutaneous tumors with anti‐PD1 antibodies (Figure 8A; Figure S8A). While anti‐PD1 monotherapy had a modest effect on control tumors, it induced significant and sustained tumor regression in mice with EEPD1‐knockdown tumors (Figure 8B,C; Figure S8B,C). The absence of significant changes in the body weight of the mice following either treatment (Figure S8D) indicates a promising safety and tolerability profile, supporting the potential of this combination strategy for clinical translation. Flow cytometric analysis of these tumors revealed that the combination treatment group had the highest infiltration of CD8^+^ T cells, which also expressed the highest levels of GZMB and IFN‐γ (Figure 8D–H; Figure S8E–J).

To confirm the critical role of these cells, we depleted CD8^+^ T cells using an anti‐CD8α antibody. This depletion partially reversed the tumor growth suppression caused by EEPD1 knockdown (Figure 8I; Figure S8K–M), confirming that CD8^+^ T cells are key mediators of the antitumor effect. In summary, our findings demonstrate that targeting EEPD1 overcomes resistance to ICB by converting a non‐immunogenic tumor into one that is highly susceptible to immune‐mediated clearance, providing a powerful strategy for combination immunotherapy in CRC (Figure 8J).

## 3 Discussion

Our study establishes the HR gatekeeper EEPD1 as a pivotal regulator of antitumor immunity in CRC. We provide a comprehensive mechanistic framework linking EEPD1‐mediated DNA repair to the immunological state of the tumor. We demonstrate that targeting EEPD1 not only directly impairs tumor cell fitness by inducing genomic instability but also converts this instability into a potent immunostimulatory signal. This signal, transduced through the cGAS‐STING‐type I IFN axis, systematically remodels the TME from a suppressive to a responsive state, ultimately sensitizing tumors to immune checkpoint blockade.

The oncogenic role of EEPD1 in gastrointestinal cancers is supported by previous reports [30, 31, 32], highlighting its critical function in the repair of damaged DNA replication forks. Notably, while acute replication fork stalling typically triggers an intra‐S phase arrest, we observed a pronounced G1/S phase arrest following EEPD1 knockdown. We attribute this to the chronic nature of the replication stress induced by sustained EEPD1 deficiency. As a critical HR gatekeeper, the prolonged loss of EEPD1 leads to the gradual accumulation of collapsed forks and under‐replicated DNA regions. Under such chronic stress, cells may bypass the immediate intra‐S checkpoint and progress through the cell cycle with unresolved lesions. Upon entering the subsequent G1 phase, these persistent damages activate the G1/S checkpoint to prevent the re‐replication of broken templates, reflecting a long‐term adaptive response to severe DNA damage. On the one hand, our findings confirm that EEPD1 depletion compromises HR repair and restricts CRC proliferation. On the other hand, we significantly extend this understanding by uncovering its profound impact on the TME. By inducing genomic instability, EEPD1 loss increases the tumor mutational burden and promotes the generation of neoantigens. This intrinsic increase in immunogenicity is coupled with the activation of the cGAS‐STING pathway, which further enhances antigen presentation via MHC‐I upregulation [41, 42, 43]. This dual effect provides a powerful one‐two punch, making tumor cells both more visible and more susceptible to immune attack.

A key advance of our study is the comprehensive characterization of the multi‐cellular immune response orchestrated by EEPD1 inhibition. While prior work on DDR and immunity has often focused on CD8^+^ T cells [25, 44, 45], we reveal a broader network of interactions. EEPD1 depletion in tumor cells promotes the maturation of DCs and skews macrophages toward a proinflammatory M1 phenotype. As professional antigen‐presenting cells, these activated myeloid cells are critical for priming and sustaining robust CD8^+^ T cell responses [46, 47, 48, 49]. Our co‐culture experiments confirm that both DCs and macrophages contribute to the heightened T cell activation and cytotoxicity observed, highlighting a coordinated immune assault triggered by a tumor‐intrinsic defect.

We further demonstrate that this immunological reprogramming is critically dependent on type I IFN signaling. By specifically blocking IFNAR1 on tumor cells prior to co‐culture, we confirmed that the observed enhancements in DC maturation, macrophage polarization, and T cell activation are driven by tumor cell‐intrinsic IFN pathway activation. This finding clarifies the mechanism, suggesting that EEPD1‐deficient tumor cells actively secrete signals that modulate and activate surrounding immune cells. Identifying these specific downstream cytokines and chemokines represents an important avenue for future investigation.

While our study robustly demonstrates the immunological effects of EEPD1 depletion, we acknowledge its reliance on preclinical models. The development of selective small‐molecule inhibitors for EEPD1 is a critical next step for clinical translation. Future work should also focus on developing nanoparticle or liposomal delivery systems for EEPD1‐siRNA or gene‐editing components to enable tumor‐specific targeting. Beyond immunotherapy, EEPD1 inhibition may sensitize tumors to chemotherapy and radiation. Finally, the identification and validation of predictive biomarkers—such as homologous recombination deficiency status or genomic instability markers—will be essential to stratify patients most likely to benefit from EEPD1‐targeted combination strategies.

Specifically, due to the current scarcity of comprehensive transcriptomic datasets from real‐world CRC patient cohorts receiving immune checkpoint blockade, we were unable to directly validate the correlation between EEPD1 expression and clinical immunotherapeutic response. Future clinical investigations incorporating paired transcriptomic and patient outcome data will be critical to fully substantiate the translational relevance of these findings in human patients.

In summary, we have identified EEPD1 as a key nexus linking DNA repair, genomic instability, and antitumor immunity in colorectal cancer. Targeting EEPD1 unleashes a powerful, multi‐faceted immune response that reverses immune tolerance and synergizes with anti‐PD1 therapy. These findings nominate EEPD1 as a compelling therapeutic target and provide a strong rationale for its clinical investigation in CRC immunotherapy.

## 4 Materials and Methods

### 4.1 Patients and Clinical Samples

Human colorectal cancer tissue specimens and paired adjacent normal tissues were obtained from patients undergoing surgical resection at The First Affiliated Hospital of Sun Yat‐sen University [IIT‐2025‐877]. All participants provided written informed consent prior to sample collection. The study was approved by the Institutional Ethics Committee of the hospital and was conducted in strict accordance with the ethical principles of the Declaration of Helsinki.

### 4.2 Antibodies and Reagents

Primary antibodies used for immunoblotting, immunofluorescence, and flow cytometry were sourced as follows: From Cell Signaling Technology (CST): anti‐cGAS (D1D3G, #79978), anti‐phospho‐STING (Ser365) (D8F4W, #72971). From Proteintech: anti‐EpCAM (#66316‐1‐Ig), anti‐cGAS (#29958‐1‐AP), anti‐TBK1 (#67211‐1‐Ig), anti‐STING (#19851‐1‐AP), anti‐GZMB (#13588‐1‐AP), anti‐EEPD1 (#24310‐1‐AP), anti‐Histone H3 (#17168‐1‐AP), anti‐GAPDH (#60004‐1‐Ig), anti‐PD‐L1(#66248‐1‐Ig). From Abcam: anti‐CD8a (#ab251596), anti‐GZMB (#ab208586), anti‐BrdU (#ab6326), anti‐phospho S386 IRF3 (ab76493). From Santa Cruz Biotechnology: anti‐dsDNA (sc‐58749). From HUABIO: custom anti‐human EEPD1 (412‐569aa). From Selleck: anti‐IRF3 (F0521).

For in vivo studies, the following antibodies were purchased from Selleck: anti‐PD1 (clone RMP1‐14), IgG2a isotype control (clone 2A3), anti‐CD8a (clone 2.43), and IgG2b isotype control (clone LTF‐2).

For flow cytometry, antibodies were purchased from BioLegend unless otherwise specified: APC‐SIINFEKL (#141605), APC‐Cy7 anti‐F4/80 (#123117), PE anti‐human HLA‐A,B,C (#311405), PE anti‐mouse H‐2Kb (#116507), BV421 anti‐mouse I‐A/I‐E (#107631), FITC anti‐CD11b (#101205), APC anti‐CD80 (#104713), PE‐Cy7 anti‐CD86 (#105013), PE anti‐CD206 (#141705), Spark Blue 550 anti‐CD3 (#100259), AF700 anti‐CD8a (#100729), BV421 anti‐FOXP3 (#126419), PE/Dazzle 594 anti‐Granzyme B (#372215), BV711 anti‐Ki‐67 (#151227), BV605 anti‐IFN‐γ (#505839), PE anti‐human CD274 (#329705), PE anti‐mouse CD274 (#124307). Viability dyes included Zombie Aqua (#423101, BioLegend) and Fixable Viability Dye eFluor 780 (#65‐0865‐14, Invitrogen).

Recombinant murine cytokines were from PeproTech: IFN‐γ (#315‐05), IL‐4 (#214‐14), M‐CSF (#315‐02), GM‐CSF (#315‐03), IL‐2 (#212‐12). Dynabeads Mouse T‐Activator CD3/CD28 were from Thermo Fisher Scientific (#11452D).

### 4.3 Cell Culture

Colorectal cancer cell lines, including HCT116 (RRID: CVCL_0291), HT29 (RRID: CVCL_0320), mouse MC38 (RRID: CVCL_B288), and non‐cancerous 293T cells (RRID: CVCL_0063) were obtained from the Cell Bank of Shanghai Institutes of Biological Sciences (Shanghai, China) in September 2021. CT26 cell line (RRID: CVCL_7254) was obtained from ATCC in September 2021. NCM460 (RRID: CVCL_0460) was obtained from Guangzhou Chengke Biotechnology Co., Ltd. (Guangzhou, China) in 2026. All cell lines were authenticated using the short tandem repeat method and tested negative for Mycoplasma. HCT116 and HT29 were cultured in Dulbecco's modified Eagle's medium (DMEM) (GIBCO) medium, while MC38 and CT26 were cultured in 1640 medium (GIBCO), both were supplemented with 10% fetal bovine serum (FBS) and 1% penicillin/streptomycin (penicillin 100 U/mL and streptomycin 10 µg/mL). Primary CD8^+^T cells, bone marrow‐derived macrophages (BMDMs), and bone marrow‐derived dendritic cells (BMDCs) were cultured in 10% FBS 1640 medium (GIBCO) containing 1% penicillin/streptomycin. CD8^+^T cells were supplemented with mouse IL‐2 (50 ng/mL) and were activated by CD3/CD28 activating dynabeads according to the needs of the experiment. BMDMs were treated with M‐CSF (20 ng/mL) while BMDCs were treated with G‐MCSF (20 ng/mL) and IL‐4 (20 ng/mL). All cell lines were authenticated by short tandem repeat (STR) fingerprinting at the Medicine Laboratory of the Forensic Medicine Department of Sun Yat‐Sen University (Guangzhou, China), and were tested to be free of mycoplasma contamination.

### 4.4 Plasmids, Virus Production, and Transfection

The lentiviral shRNAs that targeted human and murine EEPD1 were cloned into the pLVX‐puro or pLVX‐neo vector. Plasmids pLV3‐CMV‐OVAL (chicken)‐GFP‐Neo was purchased from MiaoLing Plasmids Inc (Wuhan, China). The sgRNAs that targeted murine STING were cloned into the lentiviral CRISPR/Cas9. Lentiviruses were packaged in HEK293T cells by Lipofectamine 3000 reagent (Invitrogen) according to the manufacturer's protocol and collected after concentration. Target cells were transfected with virus particles for 72 h and selected by specific antibiotics (puromycin or neomycin) or by fluorescence‐activated cell sorting (FACS). Knockdown efficiency was assessed by qRT‐PCR and western blot. The shRNA and sgRNA target sequences in this study are provided in Table S1.

### 4.5 Tissue Immunofluorescence Staining (IF) and Immunohistochemistry (IHC) Assay

IF for paraffin‐embedded specimens from either patients or mouse subcutaneous xenografts was performed in strict accordance with the protocol of the commercial tissue IF kit (Panovue Biotech, Beijing, China). Images were acquired using a Zeiss Axio Scan Z1 slide scanner (Germany). For cellular IF, cells grown on coverslips under the indicated conditions were fixed with 4% paraformaldehyde, washed with PBS, permeabilized with 0.3% Triton X‐100, and blocked with 10% BSA. Subsequent incubations with primary and secondary antibodies were carried out following the manufacturer's instructions. Imaging was performed with a Zeiss inverted fluorescence microscope. For IHC assays, serial sections of paraffin‐embedded human colon cancer specimens were separately stained with anti‐EEPD1 and anti‐CD8a antibodies. The immunostaining intensity was evaluated using either the Allred score or the H‐score system, both of which integrate the proportion of positively stained tumor cells and the average staining intensity. Cases with scores at or above the median value were classified as the EEPD1‐high group, while those with scores below the median were assigned to the EEPD1‐low group. All slides were independently assessed by at least two investigators who were blinded to the experimental groups. Each examiner randomly selected five representative fields per section for scoring, and the final score for each sample was calculated as the mean value across these five fields. The cut‐off values for classifying high vs. low protein expression were determined based on the median of the calculated scores.

### 4.6 RNA Extraction and Real‐Time Reverse Transcription‐PCR (RT‐qPCR) Analysis

Total RNA was extracted by TRIzol Plus RNA Purification Kit (Invitrogen) strictly according to the manufacturer's protocol. Extracted RNA (1 µg) was transcribed into cDNA using ChamQ Universal SYBR qPCR Master Mix according to the manufacturer's instructions (Vazyme). HiScript II Q RT SuperMix (Vazyme) kit and gene‐specific primers (sequences listed in Table S2) were applied for RT‐qPCR. Based on the threshold cycle (Ct), the relative expression level of mRNAs was calculated as 2^− [(Ct of mRNA)—(Ct of ACTB)] ^after normalization to ACTB expression.

### 4.7 Protein Extraction and Western Blotting (WB) Analysis

For cellular and tissue protein analysis, proteins were extracted from cells or tissue by a sample buffer containing protease inhibitor and conditional phosphatase inhibitors followed by a 40 kHz ultrasonic cell‐break. For cytoplasm and nucleus protein extraction, the nuclear and cytoplasmic protein extraction kit (Beyotime, P0027) was used strictly conferring to the manufacturer's protocol. The BCA Protein Assay Kit (Thermofisher) was applied for the protein concentration determination. Proteins separated by SDS‐PAGE were transferred onto PVDF membrane using electrotransfer, blocked with 5% BSA and probed with the indicated primary and secondary antibodies. The immunoblots were visualized with immobilon enhanced chemiluminescent kit (Tianya Biotech, P1034).

### 4.8 MTT Assay and Colony Formation Assay

For the MTT assay, cells (5 × 10^3^) were planted in a 96‐well plate. Measurements were conducted at 24, 48, 72, 96, 120, and 144 h. At each time point, cells were incubated with MTT solution for 4 h, followed by optical density (OD) detection at 490 nm. The growth inhibition rate was calculated as follows: (1 – OD value of the shEEPD1 group / OD value of the shNC group) × 100%. For the colony formation assay, 1000 cells were planted in a 6‐well plate, after 14 days, the cells were fixed with 4% paraformaldehyde and stained by crystal violet staining solution. The number of colonies containing over 50 cells were recorded. Experiments were performed in three biological replicates.

### 4.9 Cell Cycle Assay

Cell cycle assay was conducted according to the protocol of the cell cycle assay kit protocol (Elabscience, E‐CK‐A351). Indicated cells were collected and wash with PBS, then fixed with 75% ethanol at a temperature of 4°C overnight. RNase was applied for RNA elimination followed by an incubation of propidium iodide (PI) at room temperature in the dark for 30 mins. Cells washed with PBS twice were then analyzed with a flow cytometer CytoFLEX (Beckman Coulter). ModfitFL was applied for the analysis.

### 4.10 Animal Experiment

Six‐week‐old female mice (18‐20 g), including BALB/c nude mice, BALB/c mice, and C57BL/6 mice, were purchased from Sun Yat‐sen University animal centre. All animal experiments conducted were strictly complying with the National Institute of Health Guide for the Care and Use of Laboratory Animals. Specific pathogen‐free conditions were provided by Sun Yat‐sen University animal center throughout the entire process. All the animal experimental procedures were approved by the Institutional Animal Care and Use Committee of SYSU (approval numbers: SYSU‐IACUC‐2025‐001484, SYSU‐IACUC‐2025‐000236, and SYSU‐IACUC‐2023‐002085). For the subcutaneous xenograft model, cells (HCT116, MC38, CT26: shNC, and shEEPD1, 3 × 10^6^) were subcutaneously injected into the dorsal flank of mice. Tumor volume was measured with a vernier scale and calculated in the formula of V  =  0.5 × D × W^2^ twice a week (V for volume, D for diameter, and W for width). HCT116‐luciferase‐hygro cells (1 × 10^6^, shNC and shEEPD1) were collected and injected intravenously for the lung metastasis model or injected intrasplenically for the liver metastasis model. For the orthotopic mouse model, HCT116 cells were injected into the caecum terminus of the anesthetized mice followed by a sterile suture of the abdominal incision. Bioluminescent images were collected every 3 days. 150 mg/kg luciferin was intraperitoneally injected into each mouse before bioluminescence signal detection and analysis with Spectrum Living Image 4.2 software (Caliper Life Sciences). Mice were sacrificed as they reached the experimental endpoint, tumoral tissue was dissociated into single cells for the follow‐up flow cytometry analysis, or fixed with 4% paraformaldehyde for H&E staining, IHC staining, or IF staining as indicated. For in vivo antibody treatment, anti‐PD1, anti‐IFNAR1, and anti‐CD8a antibodies were intraperitoneally injected at a dosage of 100 µg per mouse, once every 3 days.

### 4.11 Flow Cytometry Analysis

For cell line flow cytometry analysis, cells were collected and washed with PBS twice and resuspended in 100 µl PBS. The indicated flow antibodies were added and incubated at room temperature in the dark for 30 min. Fc blocking was conducted before the incubation if necessary. The eBioscience Foxp3 / transcription factor staining buffer set (Invitrogen, 00‐5523‐00) protocol was applied for the intracellular protein staining. After all the incubation, cells were washed and resuspended with PBS and were detected by a CytoFLEX flow cytometer (Beckman Coulter). For tissue dissociation prior to the single cell flow analysis, the mouse tumor dissociation Kit (Miltenyi Biotec, 130‐096‐730) was used followed by a procedure of removing red blood cells. Then the staining protocol was the same as the cell line flow cytometry mentioned before. Data was processed via CytoExpert or FlowJo 10.8.1.

### 4.12 RNA‐Seq and Analysis

Total RNA was extracted by TRIzol reagent as mentioned in the RNA extraction method mentioned before. RNA samples were then sent to Chi Biotech (Shenzhen, China) for the subsequent RNA quality evaluation and sequencing analysis. RNA‐seq was performed on illumina platform NovaSeq xplus. Genes with Log2(fold change) > 1.5 were selected as differential genes (DEGs) for the following pathway enrichment. Gene ontology (GO) terms and GSEA gene sets were applied for the analysis.

### 4.13 Whole Exome Sequencing (WES) for Mutation Burden and Neoantigen Prediction Analysis

The WES was conducted by Chi Biotech (Shenzhen, China). Libraries were prepared by the company, and samples were then sequenced on the illumina NovaSeq xplus instrument. To identify the newly emerging variants in HCT116‐shNC and HCT116‐shEEPD1, HCT116‐parental was taken as a control. For neoantigen prediction from the variants, the somatic mutations were selected and analyzed with NetMHCPan and NetMHCIIPan software. The tumor mutation burden (TMB) was also calculated on the basis of newly emerged somatic mutations.

### 4.14 MC38‐OVA and Primary Cell Coculture

BMDCs and BMDMs were isolated from OT‐I mouse femur bone marrow and were cultivated as mentioned in the cell culture method for 6 days. Indicated MC38‐OVA cells were cocultured with BMDCs or BMDMs at a ratio of 1:1 in a 12‐well plate for 48 h. 0.4 µm transwells were used for the indirect co‐culture. After 48 h, cells were collected for flow cytometry analysis or fixed for IF staining.

### 4.15 T Cell Migration Assay

To evaluate the migratory capacity of activated OT‐I CD8^+^ T cells, 24‐well Transwell plates (5 µm) were used. Activated OT‐I CD8^+^ T cells (5 × 10^5^) suspended in 200 µL of culture medium were seeded into the upper chamber, while 5 × 10^4^ MC38‐OVA cells were plated in the lower chamber. Following a 12‐h co‐culture period, cells were harvested and subjected to flow cytometric analysis. For absolute cell quantification, a predetermined number of counting beads (BioLegend, Cat #424902) was added to each sample prior to acquisition. The migration rate was calculated using the following formula: Migration rate (%) = 100 × [(number of CD8^+^ T cells in the lower chamber/number of beads)]/[(number of CD8^+^ T cells in the input sample/number of beads)].

### 4.16 T Cell Proliferation Assay

OT‐I CD8^+^T cells were extracted from the mouse spleen and pre‐stained with CFSE (1 µM). MC38‐OVA cells were cocultured with OT‐I CD8+T cells at a ratio of 1:5. The coculture ratio of MC38‐OVA cells, BMDCs, and OT‐I CD8+T cells was 1:1:5, the coculture ratio of MC38‐OVA cells, BMDMs, and OT‐I CD8+T cells was 1:1:5, the coculture ratio of MC38‐OVA cells, BMDCs, BMDMs, and OT‐I CD8+T cells was 1:1:1:5. After a 24 h coculture, cells were collected for flow cytometry analysis as described in the protocol.

### 4.17 T Cell Intracellular Staining Assay

OT‐I CD8^+^T cells were isolated and activated with CD3/CD28 Dynabeads for 72 h followed by coculture protocols mentioned previously. The coculture lasted 6 h while BFA (3ug/mL, Invitrogen, 00‐4506‐51) was added for the last 5 h. After the coculture, cells were collected and stained with anti‐CD8a flow antibody followed by an overnight fixation and permeated by eBioscience Foxp3 / transcription factor staining buffer set (Invitrogen, 00‐5523‐00) protocol. After washing with PBS, cells were then incubated with anti‐GZMB and anti‐ki‐67 antibodies at room temperature for 30 mins. Washed and resuspend with PBS, cells were then analyzed by flow cytometry.

### 4.18 Homologous Recombination Assay

5 × 10^5^ colorectal cells were co‐transfected with 4 µg pHPRT‐DR‐GFP plasmid and 2 µg pCBASce or empty pcDNA vector, GFP expression in cells was analyzed at 48 h by flow cytometry.

### 4.19 DNA Fiber Assay

Colorectal cells were sequentially incubated with CldU (25 µM) for 20 mins, Hydroxyurea (HU, 5 µM) plus IdU (250 µM) for 20 mins and then IdU (250 µM) for 20 mins. The DNA fiber assay procedure was strictly followed according to the protocol [50]. After the incubation, cells were collected and lysed at a density of 4 × 10^5^/mL for the following spreading, fixation, and immunostaining steps. Images were obtained by a Zeiss inverted fluorescence microscope and the length of IdU and CldU was measured. The IdU/CldU ratio was calculated to reveal the cell replication fork repair capacity.

### 4.20 Cyclic GAMP (cGAMP) ELISA Assay

2 × 10^6^ colorectal cells were seeded in a 10 cm plate and cultivated to 80%–90% confluency (2–3 days of incubation) before being collected and lysed with lysis buffer. The following cGAMP detection was conducted with the 2'3'‐ cGAMP elisa kit (Thermofisher, EIAGAMP). After the antigen binding, chromogen adding, and stop solution addition, the absorbance at 450 nm was measured. Experiments were performed in three biological replicates.

### 4.21 Bioinformatic Analysis

For the TCGA‐COAD dataset immune analysis, STAR‐counts data and corresponding clinical information of COAD patients were downloaded from the TCGA database (https://portal.gdc.cancer.gov). TPM format data were extracted and normalized by the log_2_(TPM + 1) transformation. After the normalization, we used the “immunedeconv” R package that integrates the latest algorithms, including TIMER and CIBERSORT, for evaluating immune cell infiltration. The tumor immune dysfunction and exclusion (TIDE) algorithm was also applied to predict potential immune therapy responses. Statistical analysis was conducted using R software and was considered significant when the *p*‐value < 0.05. EEPD1 expression level and survival analysis in COAD patients were conducted on the GEPIA website (http://gepia.cancer‐pku.cn). The 200 genes most correlated with the shorter overall survival in COAD patients were also filtered by the GEPIA website.

### 4.22 Statistical Analysis

Except for RNA‐seq and WES analyses, all experiments were performed with at least three biologically independent samples or three biologically independent experiments unless stated otherwise. All statistical analyses were performed using GraphPad Prism version 9 (GraphPad Software, USA). A two‐tailed Student's *t*‐test was applied for comparisons between two groups. One‐way or two‐way analysis of variance (ANOVA) was used to compare multiple groups and the log‐rank test was used to compare the patient survival curves. All error bars represent mean ± SD.* p* < 0.05 was considered statistically significant.

## Author Contributions

Conceptualization: Liyun Huo, Weijing Zhang, Tianyu Tao, Methodology: Liyun Huo, Chong Wu, Xiaobo Li, Investigation: Liyun Huo, Chong Wu, Caina Ma, Visualization: Liyun Huo, Jiamin Huang, Zishuo Chen, Funding Acquisition: Weiling He, Weijing Zhang, Tianyu Tao, Project Administration: Weijing Zhang, Liyun Huo, Supervision: Tianyu Tao, Weijing Zhang, Writing – original Draft: Liyun Huo, Chong Wu, Writing – review and Editing: Tianyu Tao, Weijing Zhang, Weiling He

## Funding

This study was partially supported by the National Key Research And Development Program of China (2023YFC3402103, 2022YFC3401000), Guangdong Science and Technology Planning Program (2021B1212040017), National Natural Science Foundation of China (82403091, 82472087, 92359302, 82241231, 82030088, 82550006, U24A20740), Natural Science Foundation of Fujian Province (2024J011004), Fujian Provincial Health and Medical High‐Level Talent Team (XM050005), the Key Technologies R&D Program of Guangdong Province (2023B1111030003, 2023B1111020005), Guangdong Basic and Applied Basic Research Foundation (2024B1515040030, 2024B1515040029, 2023B111020007), the Funding by Science and Technology Projects in Guangzhou (2024A04J6549) and Shenzhen Medical Research Special Fund (C2501003).

## Conflicts of Interest

The authors declare no conflicts of interest.

## Supporting information




**Supporting File 1**: advs75054‐sup‐0001‐SuppMat.docx.

**Supporting File 2**: advs75054‐sup‐0002‐TablesS1.docx.

## Data Availability

The data that support the findings of this study are available from the corresponding author upon reasonable request.
