# Supplementary material for: EEPD1 Inhibition Unleashes Antitumor Immunity in Colorectal Cancer by Activating the cGAS‐STING Pathway
Source: Adv Sci (Weinh). 2026 Mar 30;13(34):e22826. doi: 10.1002/advs.202522826 (PMC13285135; doi:10.1002/advs.202522826)
Supplement: Supplementary file 1 — Supporting File 1: advs75054‐sup‐0001‐SuppMat.docx. [file ADVS-13-e22826-s002.docx]

**
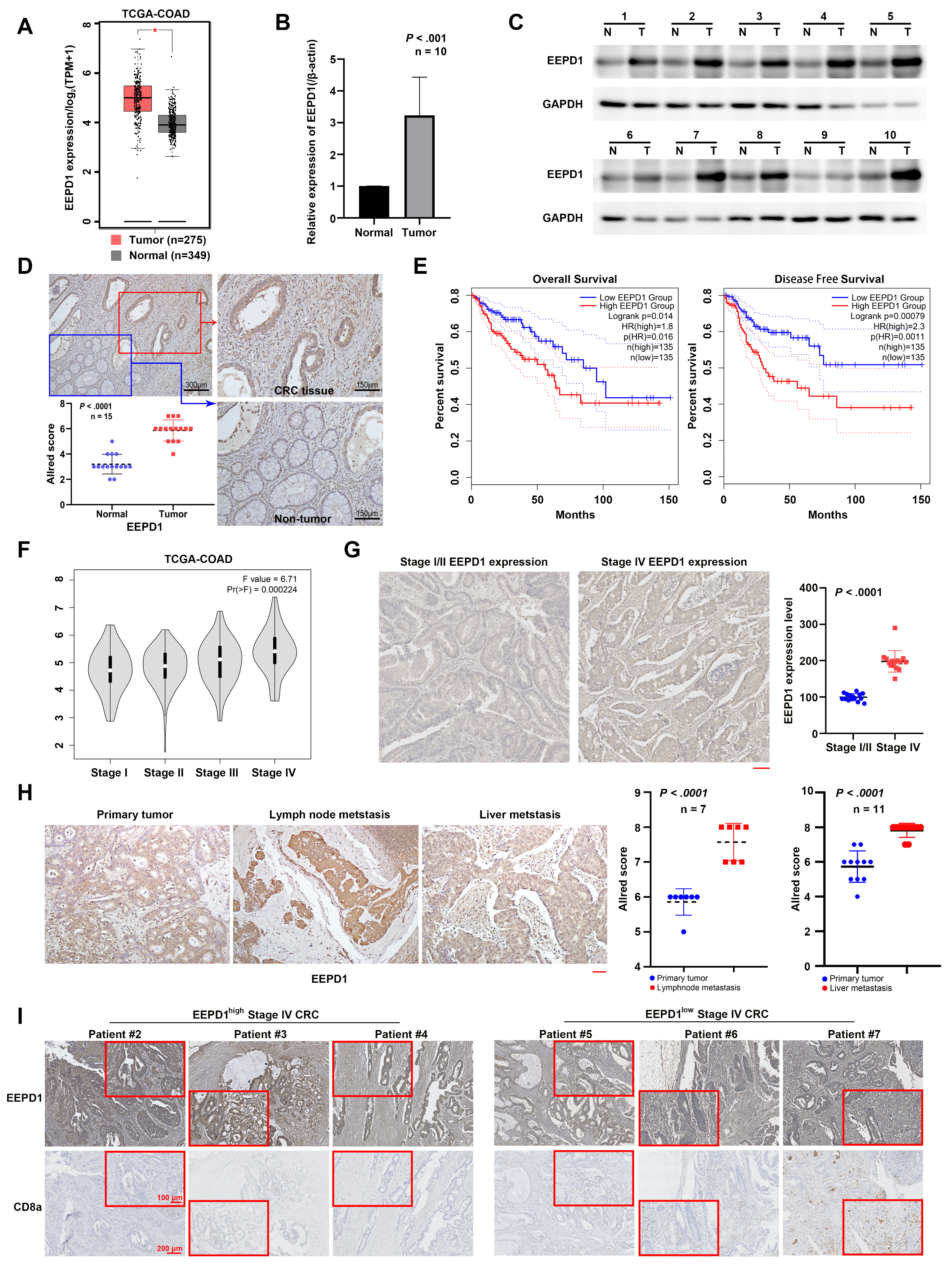
**

**Figure. S1. EEPD1 is highly expressed in advanced CRC and correlates with poor prognosis.** (A) EEPD1 mRNA expression in COAD tumor (T) versus normal (N) tissues from the GEPIA database. (B, C) RT-qPCR (B) and western blot (C) analysis of EEPD1 expression in 10 paired CRC and adjacent normal tissues. (*n* = 10 for B, C). (D) Representative IHC staining of EEPD1 in CRC and adjacent normal epithelium. (*n* = 15). (E) Kaplan-Meier analysis of overall survival (OS) and disease-free survival (DFS) in the TCGA-COAD cohort stratified by EEPD1 expression. (F) EEPD1 mRNA expression across different clinical stages in the TCGA-COAD cohort. (G) Representative IHC staining of EEPD1 in early-stage (I/II) versus advanced-stage (IV) CRC. Scale bar, 100 µm. (*n* = 10). (H) Representative IHC staining of EEPD1 in paired primary CRC tumors and corresponding lymph node (*n* = 7) or liver metastases (*n* = 11). Scale bar, 50 µm. (I) Representative immunohistochemistry (IHC) images of EEPD1 and CD8a staining on serial sections from advanced CRC patient tumors. Data are presented as mean ± SD. Statistical analysis was performed using an unpaired two-tailed Student’s t-test (A, B, D, G, H), one-way ANOVA (F) and the log-rank test (E). **P* < 0.05.

**
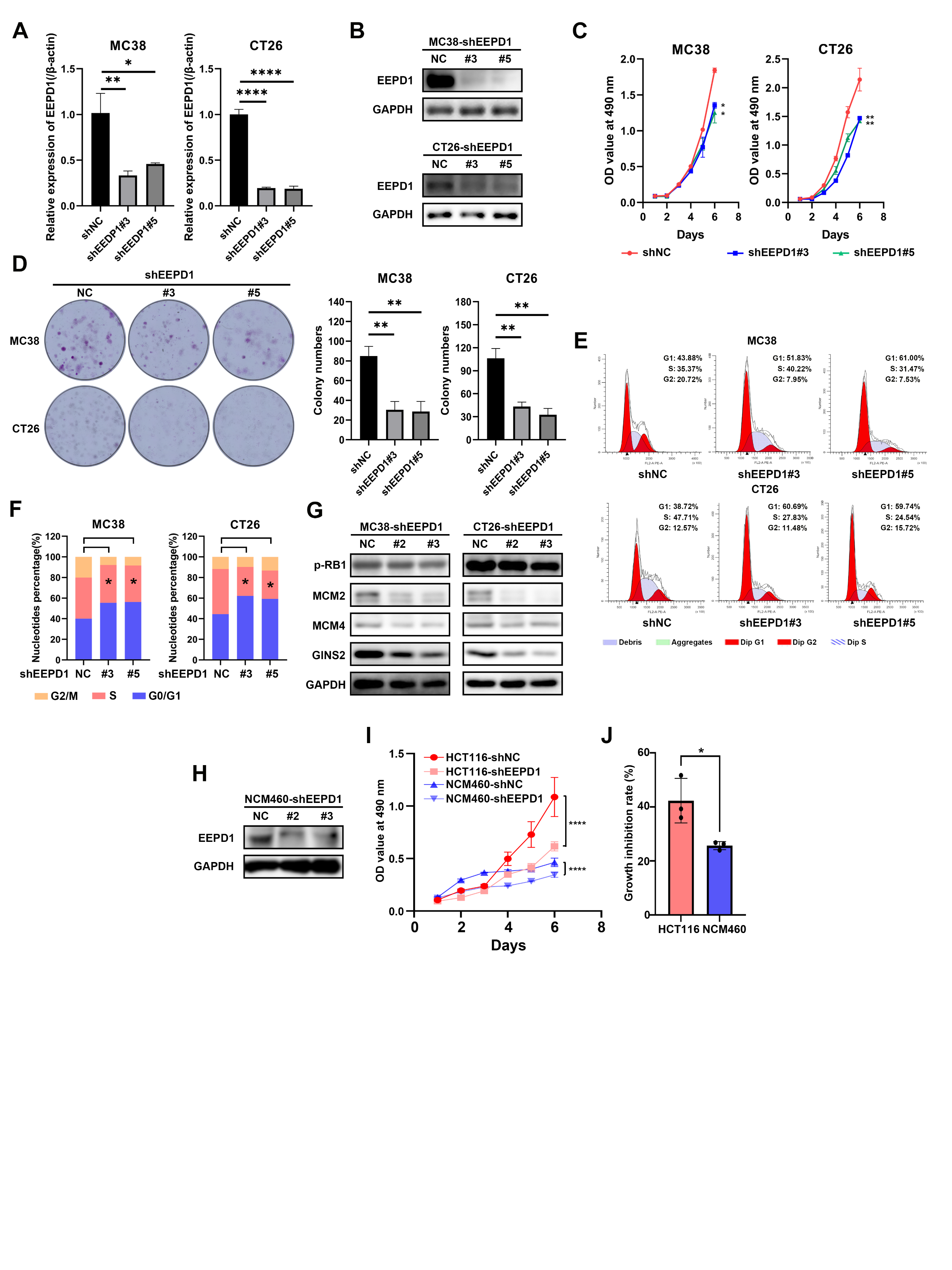
**

**Figure. S2. EEPD1 depletion induces cell cycle arrest and inhibits proliferation in murine CRC cells.** (A, B) Validation of EEPD1 knockdown in murine MC38 and CT26 CRC cells by RT-qPCR (A) and western blot (B). (C) Proliferation of shNC and shEEPD1 MC38 and CT26 cells assessed by MTT assay. (D) Colony formation capacity of shNC and shEEPD1 MC38 and CT26 cells. Representative images and quantification are shown. (E, F) Cell cycle distribution of shNC and shEEPD1 MC38 (E) and CT26 (F) cells analyzed by flow cytometry. (G) Western blot analysis of cell cycle regulatory proteins in murine CRC cell lines following EEPD1 knockdown. (H) Validation of EEPD1 knockdown in human NCM460 cells by western blot. (I) Proliferation of shNC and shEEPD1 HCT116 and NCM460 cells assessed by MTT assay. (J) Inhibition rate of shEEPD1 presented in (I). Data are presented as mean ± SD from three independent experiments (*n* = 3). Statistical analysis was performed using one-way ANOVA (A, D, F), two-way ANOVA (C, I) and unpaired two-tailed Student’s t-test (J). **P* < 0.05, ***P* < 0.01, *****P* < 0.0001.

**
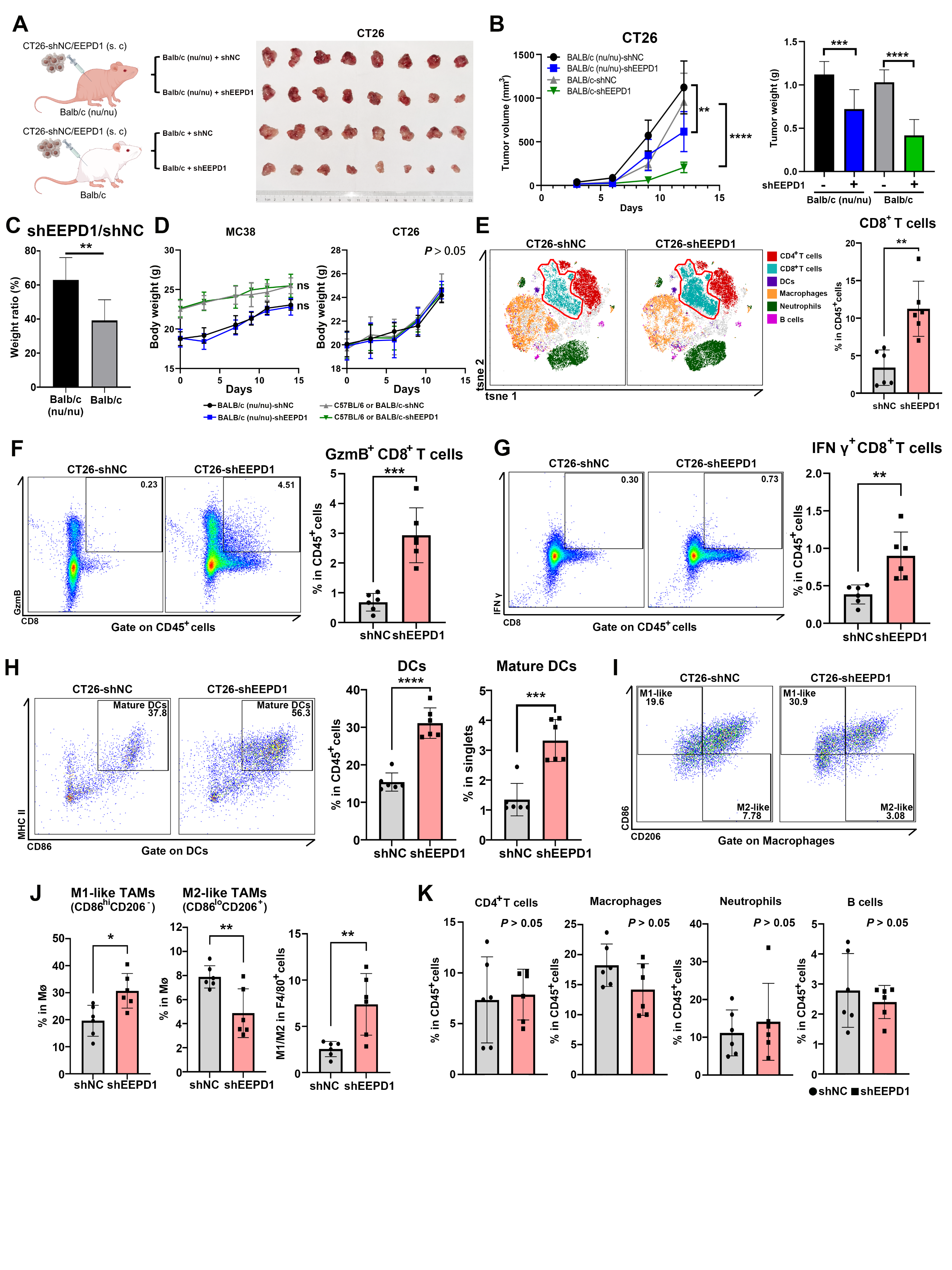
**

**Figure. S3. Targeting EEPD1 reverses the immune-tolerant microenvironment in the CT26 tumor model.** (A) Representative images of subcutaneous CT26-shNC and CT26-shEEPD1 tumors grown in immunodeficient (BALB/c nu/nu) and immunocompetent (BALB/c) mice. (B) Tumor growth curves and final tumor volumes for the experiment described in (A). (C) Tumor weight ratio (shEEPD1/shNC) in immunodeficient versus immunocompetent mice. (D) Body weight curves of mice bearing subcutaneous MC38 or CT26 tumors. (E) t-SNE visualization of the immune landscape in CT26 tumors from BALB/c mice, analyzed by spectral flow cytometry. Quantification of the CD8⁺ T cell population is shown. (F, G) Flow cytometric analysis of effector molecule expression in tumor-infiltrating CD8⁺ T cells. Representative plots and quantification of GZMB⁺ (F) and IFN-γ⁺ (G) CD8⁺ T cells. (H) Analysis of dendritic cell maturation. Representative plots and quantification of mature DCs (CD11c⁺MHC-II⁺CD86⁺). (I, J) Analysis of macrophage polarization, showing the M1/M2 ratio. (K) Quantification of tumor-infiltrating CD4⁺ T cells, total macrophages, neutrophils, and B cells. Data are presented as mean ± SD (*n* = 8 mice per group for A-D, *n* = 6 for E-K). Statistical analysis was performed using two-way ANOVA (B, D, tumor volume and body weight curve), one-way ANOVA (B, tumor weight), and an unpaired two-tailed Student’s t-test (C, E-K). **P* < 0.05, ***P* < 0.01, ****P* < 0.001, *****P* < 0.0001.

**
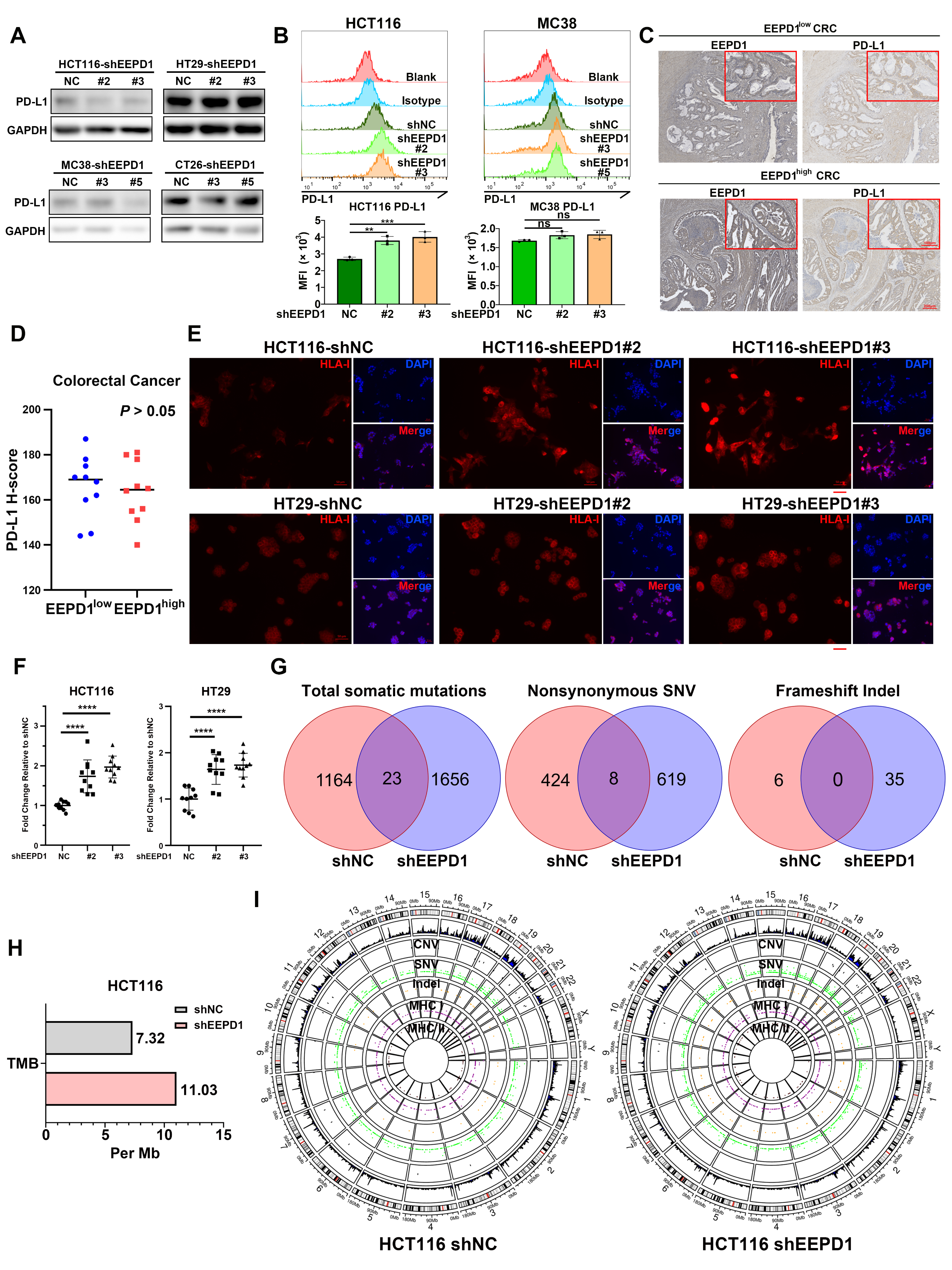
**

**Figure. S4. EEPD1 depletion enhances MHC-I expression and increases tumor mutation burden.** (A) Western blot analysis of PD-L1 protein levels in HCT116, HT29, MC38, CT26 cells. (B) Flow cytometric analysis of surface PD-L1 expression on HCT116 and MC38 CRC cell lines (*n* = 3). (C) Representative immunohistochemistry (IHC) images of EEPD1 and PD-L1 staining on serial sections from CRC patient tumors (*n* = 10), Scale bars, 200 µm and 100 µm. (D) Quantification of PD-L1 staining intensity presented in (C). (E) Representative immunofluorescence images showing HLA-I protein expression (green) in shNC and shEEPD1 HCT116 and HT29 cells. DAPI (blue) stains nuclei. Scale bar, 20 µm. (F) Relative quantification of immunoflourescence presented in (E) (*n* = 10). (G) Venn diagrams showing the overlap and number of total somatic mutations, nonsynonymous single nucleotide variants (SNVs), and frameshift indels identified by WES. (H) Tumor mutational burden (TMB) calculated from WES data, expressed as mutations per megabase. (I) Circos plots illustrating the genomic landscape of HCT116-shNC and shEEPD1 cells. Tracks from outer to inner represent: human karyotype, sequencing coverage, copy number variation (CNV), somatic SNVs, somatic indels, and predicted high-affinity MHC-I and MHC-II binding peptides. Data are presented as mean ± SD. Statistical analysis was performed using one-way ANOVA (B, F) and unpaired two-tailed Student’s t-test (D). ***P* < 0.01, ****P* < 0.001, *****P* < 0.0001.

**
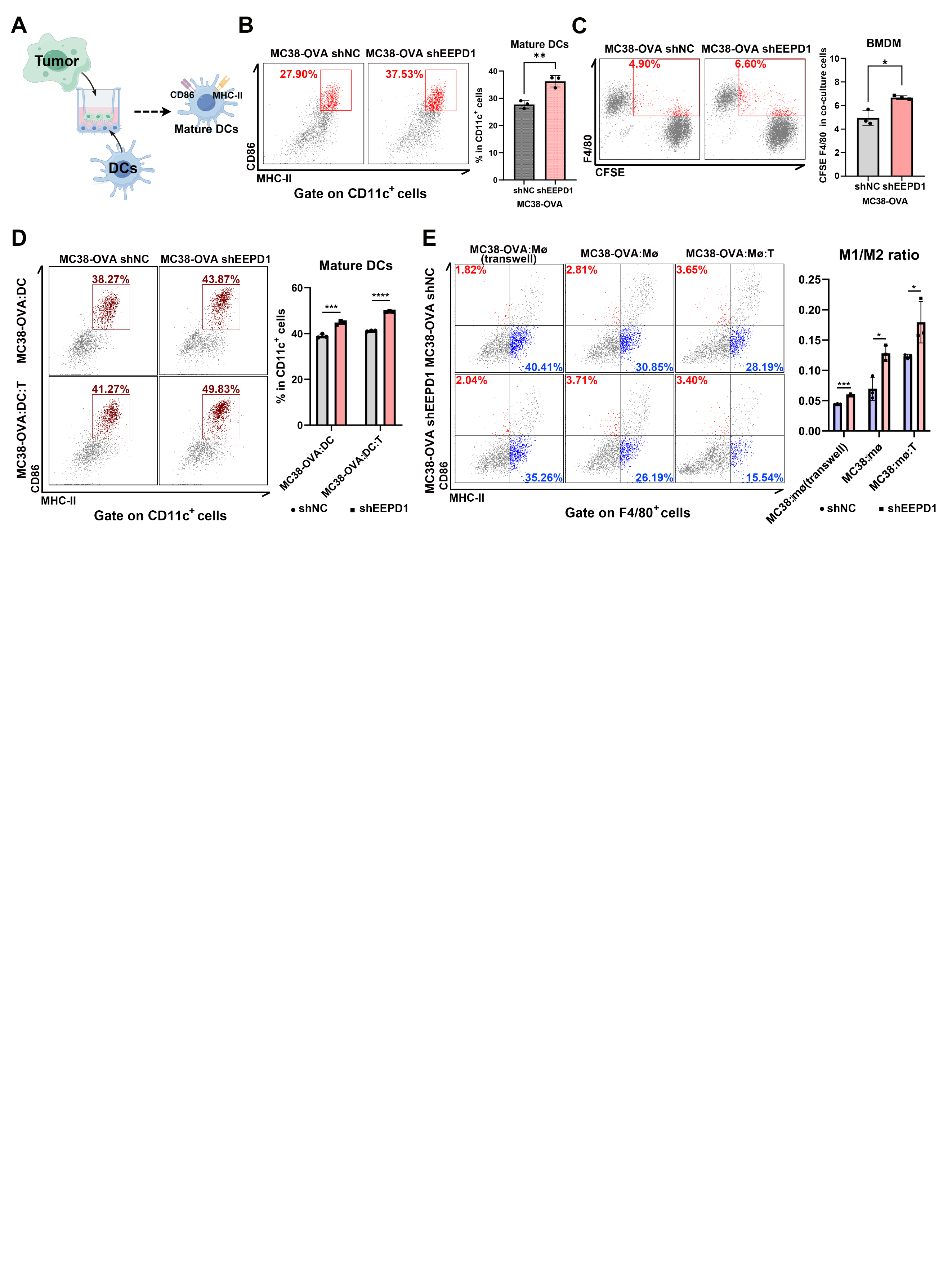
**

**Figure. S5. EEPD1 loss promotes DC maturation and macrophage phagocytosis, with evidence of a positive feedback loop.** (A, B) DC maturation assay using a transwell system. BMDCs were indirectly co-cultured with MC38-OVA cells for 48 hours. Representative plots (A) and quantification (B) of mature DCs (CD11c⁺MHC-II⁺CD86⁺). (C) Flow cytometric quantification of macrophage phagocytosis of CFSE-labeled tumor cells (F4/80⁺CFSE⁺). (D) DC maturation in the presence or absence of CD8⁺ T cells. BMDCs were co-cultured with MC38-OVA cells alone or with both tumor cells and OT-I CD8⁺ T cells. (E) Macrophage polarization in the presence or absence of CD8⁺ T cells. BMDMs were co-cultured with MC38-OVA cells alone or with both tumor cells and OT-I CD8⁺ T cells. The M1/M2 ratio is shown. Data are presented as mean ± SD from three independent experiments (*n* = 3). Statistical analysis was performed using an unpaired two-tailed Student’s t-test. **P* < 0.05, ***P* < 0.01, ****P* < 0.001, *****P* < 0.0001.

**
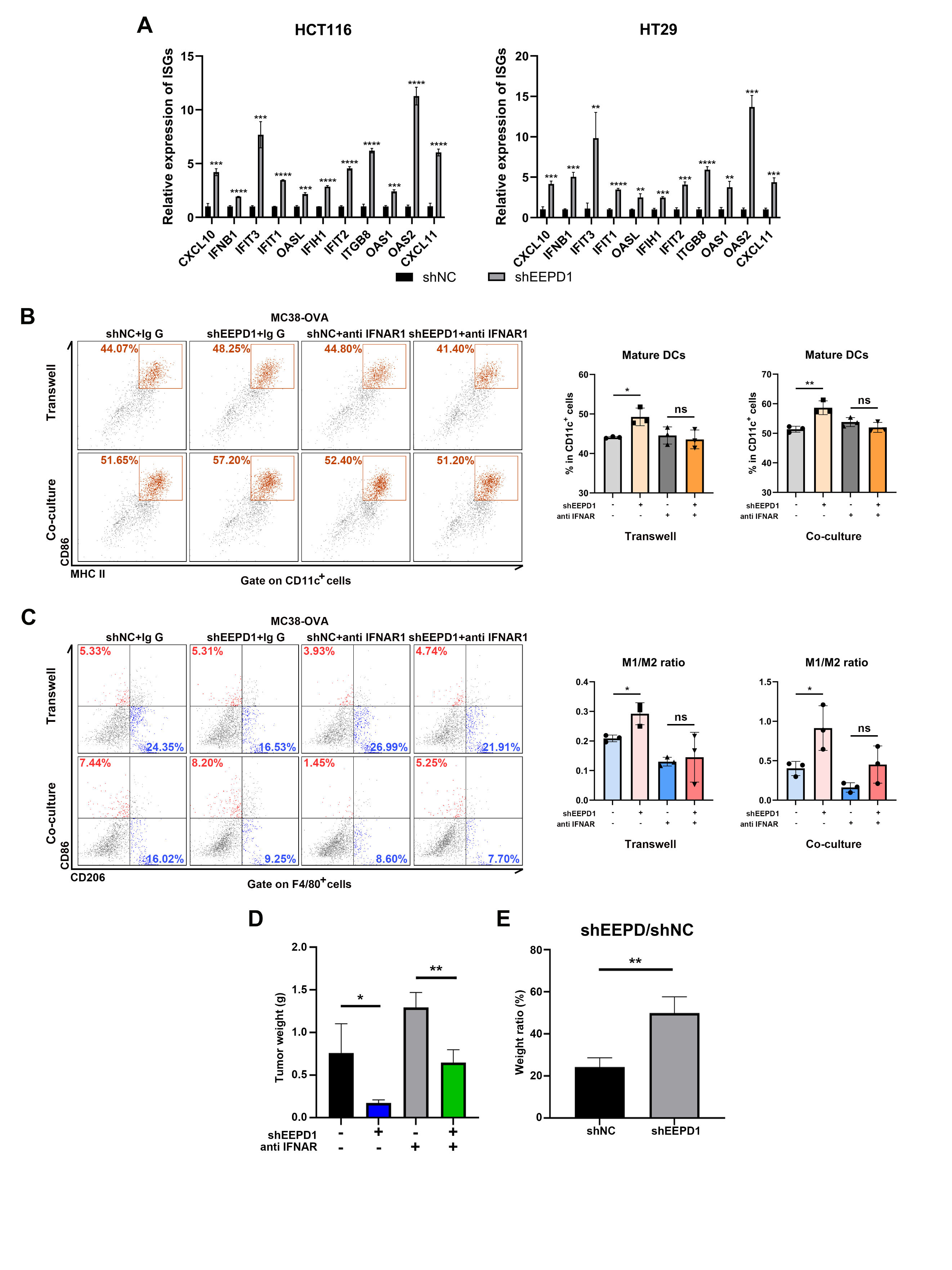
**

**Figure. S6. Tumor cell-intrinsic type I IFN signaling mediates the immunomodulatory effects of EEPD1 depletion.** (A) RT-qPCR analysis of ISG expression in EEPD1-knockdown HCT116 and HT29 cells. (B) DC maturation assay. BMDCs were co-cultured with MC38-OVA cells pre-treated with anti-IFNAR1 or isotype control. (C) Macrophage polarization assay. BMDMs were co-cultured with MC38-OVA cells pre-treated with anti-IFNAR1 or isotype control. The M1/M2 ratio is shown. (D, E) In vivo IFNAR1 blockade experiment. Final tumor weights (D) and tumor weight ratio (shEEPD1/shNC) (E) from the experiment shown in Fig. 6I. Data are presented as mean ± SD (*n* = 3 for A-C; *n* = 4 for D, E). Statistical analysis was performed using an unpaired two-tailed Student’s t-test (A, E) and one-way ANOVA (B, C, D). **P* < 0.05, ***P* < 0.01, ****P* < 0.001, *****P* < 0.0001.

**
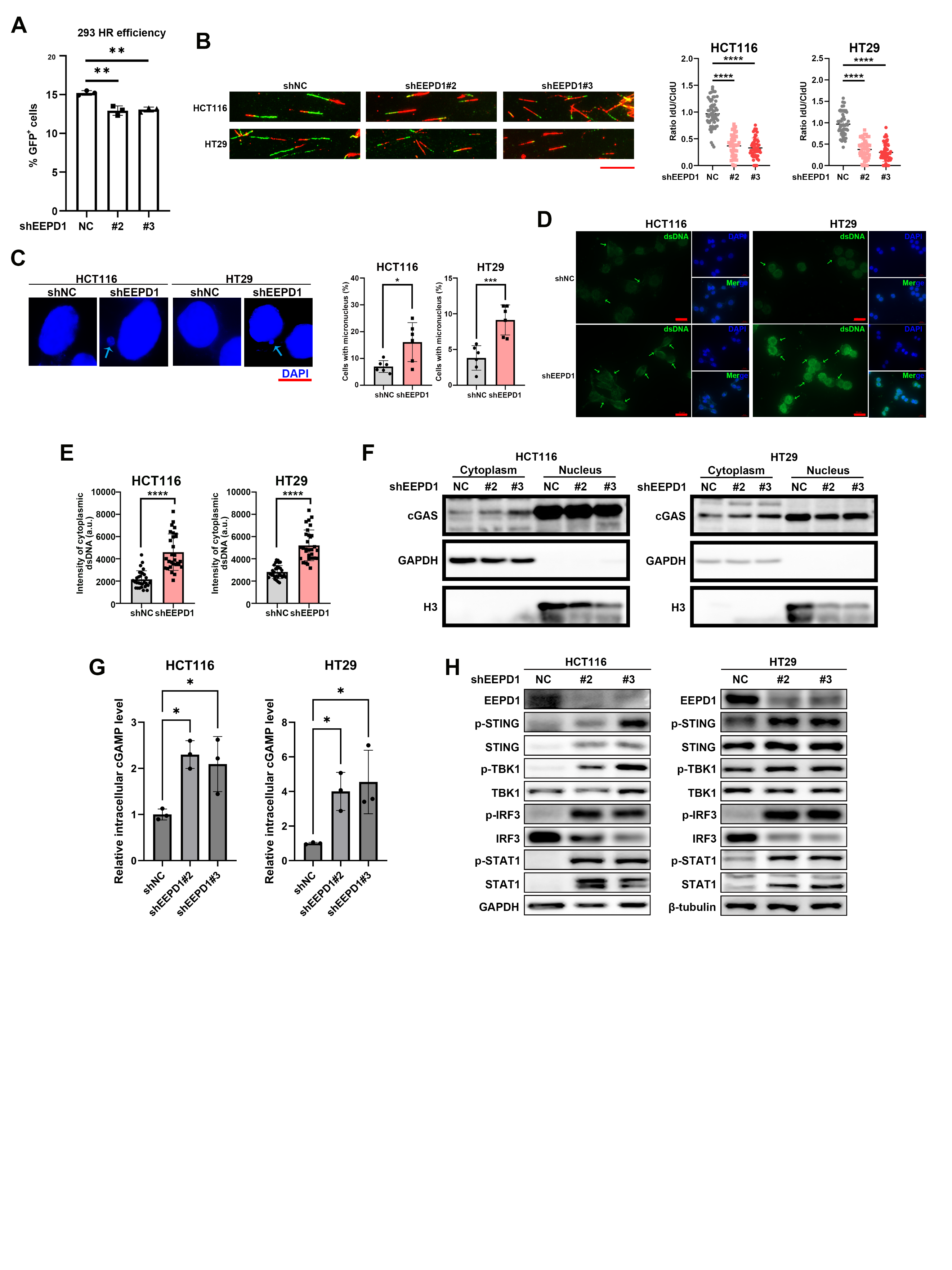
**

**Figure. S7. EEPD1 depletion in human CRC cells induces genomic instability and activates the cGAS-STING pathway.** (A) Homologous recombination (HR) repair efficiency assessed in HEK293T cells using the DR-GFP reporter assay. (B) DNA fiber assay in HCT116 and HT29 cells. Representative images and quantification of the IdU/CldU tract length ratio are shown. Scale bar, 10 µm. (C) DAPI staining showing micronucleus formation in EEPD1-knockdown HCT116 and HT29 cells. Scale bar, 10 µm. (D, E) Immunofluorescence staining (D) and quantification (E) of cytosolic dsDNA in EEPD1-knockdown human CRC cells. Scale bar, 20 µm. (F) Western blot analysis of cGAS in cytoplasmic and nuclear fractions of HCT116 and HT29 cells. (G) ELISA quantification of cGAMP levels in HCT116 and HT29 cell lysates. (H) Western blot analysis of key cGAS-STING pathway proteins in HCT116 and HT29 cells. Data are presented as mean ± SD from three independent experiments (*n* = 3 for A, G; *n* = 60 for B; *n* =6 for C; *n* = 30 for D). Statistical analysis was performed using one-way ANOVA (A, B, G) and an unpaired two-tailed Student’s t-test (C, E). **P* < 0.05, ***P* < 0.01, ****P* < 0.001, *****P* < 0.0001.

**
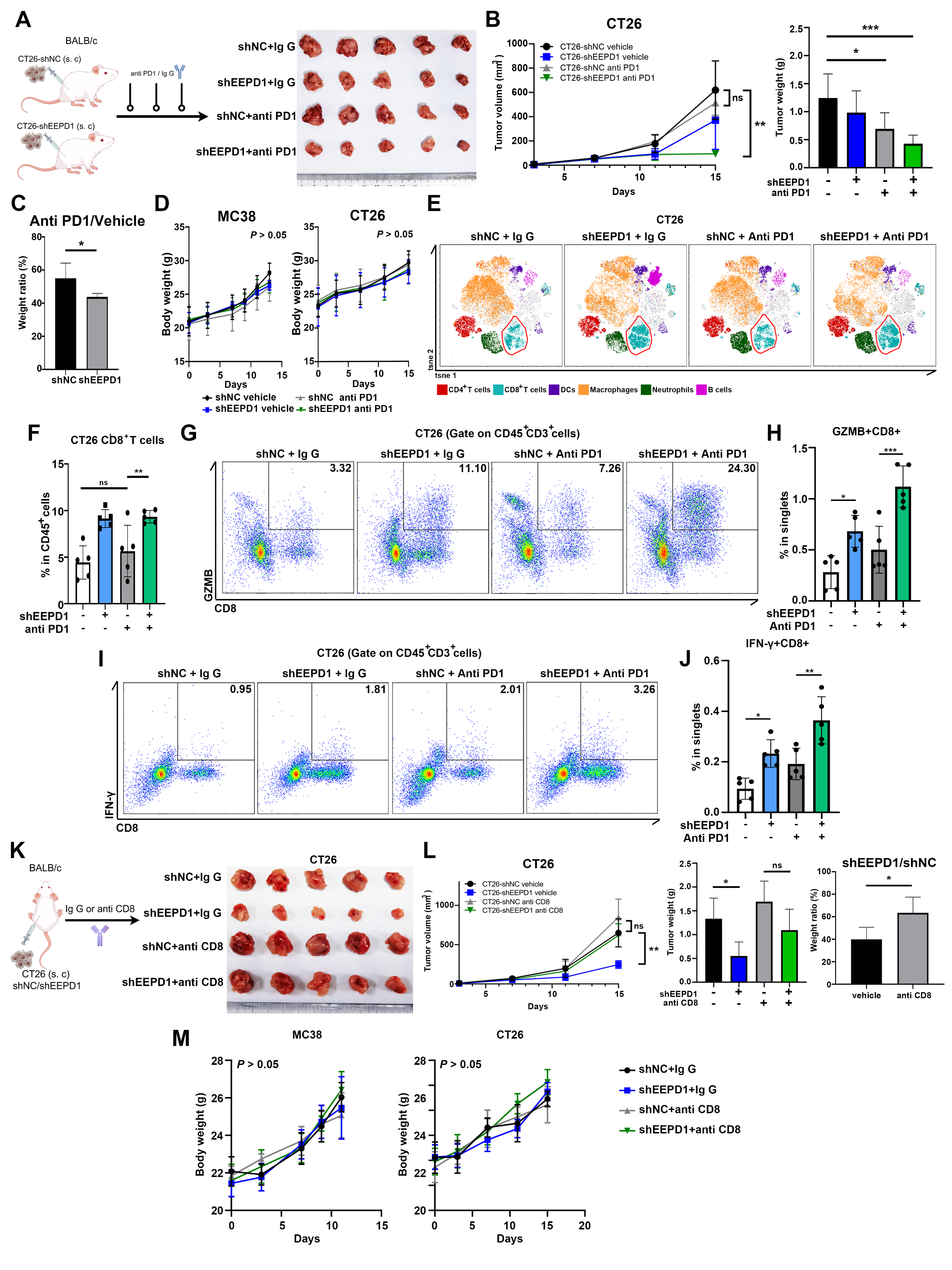
**

**Figure. S8. Targeting EEPD1 synergizes with anti-PD1 immunotherapy in the CT26 model.** (A-C) BALB/c mice bearing subcutaneous CT26 tumors were treated with anti-PD1 or isotype control antibody. (A) Representative tumor images at the endpoint. (B) Tumor growth curves and final tumor volumes. (C) Tumor weight ratio (anti-PD1/IgG). (D) Body weight curves of mice bearing subcutaneous MC38 or CT26 tumors and anti PD-1 antibody. (E, F) Flow cytometric (E) and quantification of tumor-infiltrating CD8⁺ T cells (F). (G-I) Analysis of CD8⁺ T cell effector function. Representative plots and quantification of GZMB⁺ (G, H) and IFN-γ⁺ (I, J) CD8⁺ T cells. (K, L) CD8⁺ T cell depletion experiment in the CT26 model. (K) Tumor images. (L) Tumor growth curves, final tumor volumes and final tumor weight ratio (shEEPD1/shNC). (M) Body weight curves of mice bearing subcutaneous MC38 or CT26 tumors and anti-CD8 antibody. Data are presented as mean ± SD (*n* = 5 mice per group). Statistical analysis was performed using two-way ANOVA (B, D, L, M), one-way ANOVA (B, F, H, J, L), and an unpaired two-tailed Student’s t-test (C, L). **P* < 0.05, ***P* < 0.01, ****P* < 0.001.
