# Supplementary material for: EEPD1 Inhibition Unleashes Antitumor Immunity in Colorectal Cancer by Activating the cGAS‐STING Pathway
Source: Adv Sci (Weinh). 2026 Mar 30;13(34):e22826. doi: 10.1002/advs.202522826 (PMC13285135; doi:10.1002/advs.202522826)
Supplement: Supplementary file 2 — Supporting File 2: advs75054‐sup‐0002‐TablesS1.docx. [file ADVS-13-e22826-s001.docx]

**Table S1.** **Oligos used for knockdown or knockout genes.**

| Name | Sequence (5’-3’) |
| --- | --- |
| Human EEPD1 sh2 | TCGTGGAGTACCGAGAGTATA |
| Human EEPD1 sh3 | GACAACATCTGGATCAGTAAA |
| Mouse EEPD1 sh3 | GCATCGTGGAATACCGAGAAT |
| Mouse EEPD1 sh5 | AGATTCAAGGTGGGAAGTAAT |
| Mouse STING sg | CGGCAGTTATTTCGAGACTC |

**Table S2. Sense and antisense primers used for qRT-PCR.**

| Name | Sequence (5’-3’) |
| --- | --- |
| Human EEPD1 sense | CTGGGTATGCAGGATTCCTATGG |
| Human EEPD1 antisense | GGTCGTGACTTCCCACCTTG |
| Mouse EEPD1 sense | GGCTGCCATCGCTCTATCC |
| Mouse EEPD1 antisense | TAGCCGCTCCTGATTCACCA |
| Human ACTB sense | CATGTACGTTGCTATCCAGGC |
| Human ACTB antisense | CTCCTTAATGTCACGCACGAT |
| Mouse ACTB sense | GGCTGTATTCCCCTCCATCG |
| Mouse ACTB antisense | CCAGTTGGTAACAATGCCATGT |
| Human CXCL10 sense | GTGGCATTCAAGGAGTACCTC |
| Human CXCL10 antisense | TGATGGCCTTCGATTCTGGATT |
| Human IFNB1 sense | ATGACCAACAAGTGTCTCCTCC |
| Human IFNB1 antisense | GGAATCCAAGCAAGTTGTAGCTC |
| Human IFIT3 sense | TCAGAAGTCTAGTCACTTGGGG |
| Human IFIT3 antisense | ACACCTTCGCCCTTTCATTTC |
| Human IFIT1 sense | TTGATGACGATGAAATGCCTGA |
| Human IFIT1 antisense | CAGGTCACCAGACTCCTCAC |
| Human OASL sense | CTGATGCAGGAACTGTATAGCAC |
| Human OASL antisense | CACAGCGTCTAGCACCTCTT |
| Human IFIH1 sense | TCGAATGGGTATTCCACAGACG |
| Human IFIH1 antisense | GTGGCGACTGTCCTCTGAA |
| Human IFIT2 sense | AAGCACCTCAAAGGGCAAAAC |
| Human IFIT2 antisense | TCGGCCCATGTGATAGTAGAC |
| Human ITGB8 sense | ACCAGGAGAAGTGTCTATCCAG |
| Human ITGB8 antisense | CCAAGACGAAAGTCACGGGA |
| Human OAS1 sense | TGTCCAAGGTGGTAAAGGGTG |
| Human OAS1 antisense | CCGGCGATTTAACTGATCCTG |
| Human OAS2 sense | CTCAGAAGCTGGGTTGGTTTAT |
| Human OAS2 antisense | ACCATCTCGTCGATCAGTGTC |
| Human CXCL11 sense | GACGCTGTCTTTGCATAGGC |
| Human CXCL11 antisense | GGATTTAGGCATCGTTGTCCTTT |
| Mouse OASL2 sense | TTGTGCGGAGGATCAGGTACT |
| Mouse OASL2 antisense | TGATGGTGTCGCAGTCTTTGA |
| Mouse IRF1 sense | ATGCCAATCACTCGAATGCG |
| Mouse IRF1 antisense | CCTGCTTTGTATCGGCCTGT |
| Mouse IFIH1 sense | ATGGACGCAGATGTTCGTGG |
| Mouse IFIH1 antisense | TCCCTTCTCGAAGCAAGTGTC |
| Mouse IFI44 sense | AACTGACTGCTCGCAATAATGT |
| Mouse IFI44 antisense | GTAACACAGCAATGCCTCTTGT |
| Mouse IFI27 sense | TTCCCCCATTGGAGCCAAG |
| Mouse IFI27 antisense | AGGCTGCAATTCCTGAGGC |
| Mouse CCL5 sense | GCTGCTTTGCCTACCTCTCC |
| Mouse CCL5 antisense | TCGAGTGACAAACACGACTGC |
| Mouse CXCL10 sense | CCAAGTGCTGCCGTCATTTTC |
| Mouse CXCL10 antisense | GGCTCGCAGGGATGATTTCAA |
